# A Regiospecific Co-Assembly Method to Functionalize Ordered Mesoporous Metal Oxides with Customizable Noble Metal Nanocrystals

**DOI:** 10.1021/acscentsci.4c01592

**Published:** 2024-11-21

**Authors:** Jichun Li, Lingxiao Xue, Yu Deng, Xiaowei Cheng, Junhao Ma, Wenhe Xie, Meihua Chen, Yonghui Deng

**Affiliations:** † Department of Chemistry, Shanghai Stomatological Hospital & School of Stomatology, State Key Laboratory of Molecular Engineering of Polymers, Shanghai Key Laboratory of Molecular Catalysis and Innovative Materials, Fudan University, Shanghai 200433, P. R. China; ⊥ State Key Laboratory for Modification of Chemical Fibers and Polymer Materials, College of Materials Science and Engineering, Donghua University, Shanghai 201620, P. R. China; ‡ School of Materials Science and Engineering, Nanyang Technological University, Singapore 639798, Singapore; ∥ State Key Lab of Transducer Technology, Shanghai Institute of Microsystem and Information Technology, Chinese Academy of Sciences, Shanghai 200050, P. R. China

## Abstract

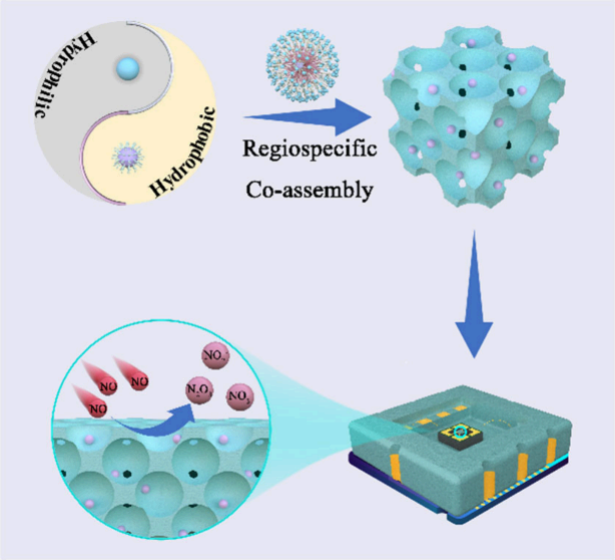

An efficient regiospecific co-assembly (RSCA) strategy is developed for general synthesis of mesoporous metal oxides with pore walls precisely decorated by highly dispersed noble metal nanocrystals with customized parameters (diameter and composition). It features the rational utilization of the specific interactions between hydrophilic molecular precursors, hydrophobic noble metal nanocrystals, and amphiphilic block copolymers, to achieve regiospecific co-assembly as confirmed by molecular dynamics simulations. Through this RSCA strategy, we achieved a controllable synthesis of a variety of functional mesoporous metal oxide composites (e.g., WO_3_, ZrO_2_, TiO_2_) with in-pore walls precisely decorated by various noble metal nanocrystals of tailored components (Au, Ag, Pt, Pd and their nanoalloys) and sizes (3.0–8.5 nm). As an example, the obtained mesoporous 0.5-Ag/WO_3_ material has a highly interconnected mesoporous structure and uniform 6.5 nm Ag nanocrystals confined in the mesopores, showing superior NO sensing performances with high sensitivity, good selectivity, and stability at low working temperature (127 °C). *In situ* spectroscopy study indicates that the NO sensing process involves a unique gas–solid reaction, where NO molecules are converted into chemisorbed NO_*x*_ species over the sensitive materials, inducing a remarkable change of resistance and outputting a dramatic response signal.

## Abstract 2

A facile regiospecific co-assembly strategy enables the obtained mesoporous WO_3_ materials with precisely decorated Ag nanocrystals and rich oxygen vacancies to show superior NO sensitivity.

## Introduction

Metal oxides with sufficient active sites and low cost have been deemed as attractive materials for chemical sensing and catalysis, while poor selectivity and high working temperature hinder their practical applications.^1,2^ Decorating metal oxides with noble metals is an efficient way to improve the selectivity and lower the working temperature.^3,4^ Besides, noble metals/metal oxides (NMs/MOs) interfaces can make extra contributions to high performance due to their extraordinary chemical and physical characteristics, including localized surface plasmon resonance effect,^5,6^ strong metal–support interaction,^7−11^ and molecule spillover effect.^12,13^ Despite the outstanding features, some challenges still remain for the synthesis of NMs/MOs nanocomposites for diverse applications. Conventional synthesis methods of NMs/MOs composites usually result in NMs/MOs with low specific surface area, poor spatial distribution, and uncontrollable composition and size of noble metal nanoparticles.^14−16^ Additionally, owing to the high surface energy, noble metal nanoparticles tend to aggregate during applications especially at high temperatures, which is unfavorable to maintain the active NMs/MOs interfaces and their application performance in various fields, such as heterogeneous catalysis and chemical sensing.^17−19^ Nowadays, tremendous efforts have been devoted to exploring nanostructured NMs/MOs composites with tailored compositions and structures to achieve high application performance.^20−24^ Rational construction of mesoporous metal oxide frameworks is emerging as an effective approach to realize high catalytic efficiencies of NMs/MOs because of the unique mesostructure, rich interfaces, and high porosity.^25−33^

Mesoporous structures have interconnected uniform mesopores (2–50 nm) that can offer channels for the transport of gas molecules dominated by Knudsen diffusion.^34,35^ More importantly, they possess high specific surface area and abundant active sites for the adsorption and catalytic conversion of target molecules, which are highly desired in heterogeneous catalysis and chemical sensing. Furthermore, the well-connected mesoporous frameworks provide confined nanospace for stable immobilization of functional noble metal nanoparticles, which can inhibit their migration and sintering.^36−39^ Various noble metals and alloys, such as Ag, Pd, Au, Pt, and Au–Pd alloy, were introduced to construct mesoporous NMs/MOs composites via different methods.^40−44^ Post-loading method that involves incipient-wetness impregnation and post-reduction process is a common approach to achieve noble metals decoration, while the random deposition and growth of noble metal nanoparticles in the opening of the pores may result in mesopores blocking.^45^ Although one-pot synthesis of mesoporous NMs/MOs with molecular noble metal precursors could prevent from blocking, it involves with both noble metal salts and metal oxide precursors co-assembling with amphiphilic block copolymer within the hydrophilic region of template micelles, which may cause the two components to interfere with each other, resulting in phase separation and affecting the formation of mesoscopic structures; and it still requires post-reduction treatment, making it difficult to control the composition and size of noble metal nanoparticles.^46^ It is highly desired yet challenging to precisely and controllably deposit certain-sized noble metal nanoparticles with tailored compositions on the inner wall of mesoporous metal oxides with abundant NMs/MOs interfaces for applications involving host–guest interactions, such as gas sensing and heterogeneous catalysis.

Herein, a regiospecific co-assembly (RSCA) method was developed to precisely deposit noble metal nanocrystals (NCs) with customizable sizes and compositions on the inner surface of mesoporous metal oxides matrixes. In this method, diverse pre-synthesized uniform hydrophobic noble metal nanocrystals and hydrophilic molecular precursors were utilized for the first time to co-assemble with amphiphilic diblock copolymer poly(ethylene oxide)-*block*-polystyrene (PEO-*b*-PS) via hydrophobic and coordination interaction, respectively. During the co-assembly process, different interactions enable the hydrophilic precursors and hydrophobic metal NCs confined in different regions of the micelles formed by the microphase separation of PEO-*b*-PS copolymers. Specifically, the hydrophobic metal NCs are encapsulated in the hydrophobic PS cores, while hydrophilic precursors of MOs are distributed around the hydrophilic PEO shells, refraining from the potential mutual interference between different precursors in the same region. Besides, the PS regions of template diblock copolymers can be derived into the mesopores of materials after calcination, thus, the size-customized nanocrystals (e.g., 3.0 nm Pt, 6.5 nm Ag and 8.5 nm Au) surrounded by PS segments can be precisely anchored in mesopores and fully exposed after templates removal. Moreover, by modulating NMs with different compositions and ratios during pre-synthesis, component-tailored nanocrystal (e.g., Au_2_Pd_1_ and Au_1_Pd_1_ alloy nanocrystals) modified mesoporous metal oxides can be readily synthesized. As a proof of concept, customized Ag nanocrystals of 6.5 nm functionalized mesoporous tungsten oxides (mesoporous 0.5-Ag/WO_3_ with 0.5 wt.% Ag content) with a specific surface area of 47.8 m^2^/g, uniform large mesopores (∼35.0 nm), crystalline WO_3_ frameworks, and monodispersed Ag nanocrystals confined in the mesopores were synthesized via this RSCA method. By virtue of the high catalytic activity of Ag NCs and abundant oxygen vacancies, the obtained mesoporous 0.5-Ag/WO_3_ materials exhibited excellent gas sensing performance (response value of 250 to 25 ppm of NO) at a relatively low working temperature of 127 °C. Furthermore, a mesoporous 0.5-Ag/WO_3_ based MEMS sensor with high integration displayed a good linear relationship and repeatability between the sensing responses and NO concentrations in the range of 1–25 ppm. Density functional theory (DFT) calculations and electron paramagnetic resonance (EPR) analysis suggest that the decoration of 6.5 nm Ag NCs with high dispersion is beneficial for the chemisorption and oxidization of NO molecules during the sensing process. *In situ* diffuse reflectance Fourier transform infrared spectroscopy (*in situ* DRIFTS) study revealed that NO molecules were oxidized into chemisorbed NO_*x*_ species (NO_2_^–^, N_2_O_3_, and NO_3_^–^), and thus accounts for the enhanced NO sensing performance which favors their applications in different fields.

## Results and Discussion

### Synthesis and Characterizations of Materials

**Scheme 1:**
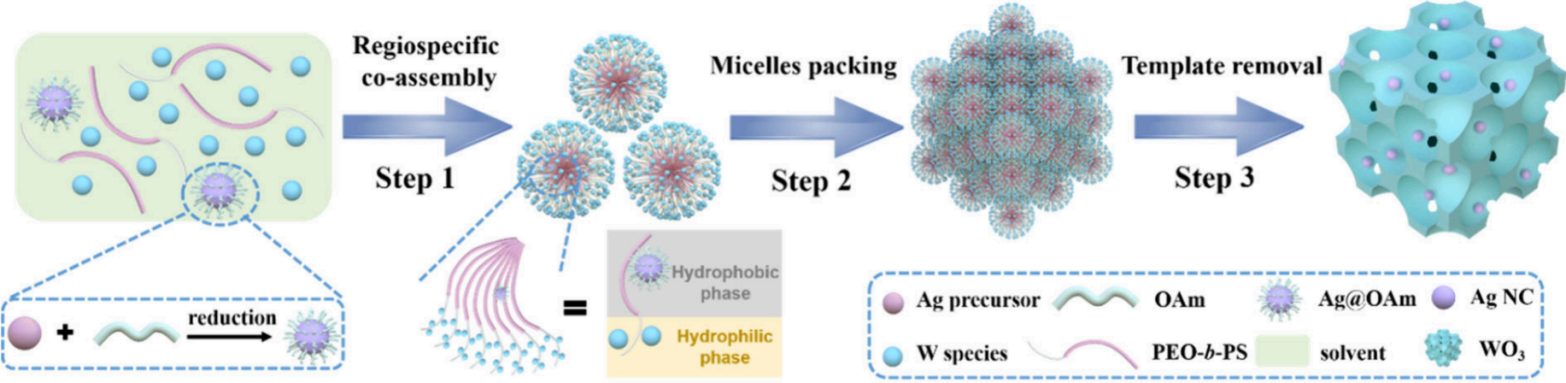
Illustration of the Regiospecific Co-Assembly in Tetrahydrofuran/Ethanol Solution for the Synthesis of Mesoporous Noble Metal/Metal Oxide (e.g., Ag/WO_3_) Composites

The RSCA strategy for the synthesis of noble nanocrystal-functionalized mesoporous metal oxides, i.e., NMs/MOs composites, is shown in Scheme 1. Amphiphilic PEO-*b*-PS copolymers were used as the structure-directing agent to co-assemble with pre-synthesized hydrophobic metal NCs and hydrophilic metal oxide precursors. Taking the synthesis of mesoporous Ag/WO_3_ as an example, PEO-*b*-PS copolymers interact with oleylamine (OAm)-capped Ag NCs (∼6.5 nm, Figure S1) via hydrophobic interaction between PS segments and OAm, while the hydrophilic PEO segments interact with acetylacetone-stabilized tungsten species by coordination bonds. First, a volatile solution containing precursors was prepared and allowed to evaporate solvents at ambient temperature, and uniform spherical composite micelles can be formed without phase separation once the concentration of PEO-*b*-PS achieves the critical micelles concentration.^25^ Owing to the specific interactions, Ag NCs could be included in the hydrophobic PS core and acetylacetone-stabilized tungsten species were associated with PEO shell of the composite micelles (regiospecific co-assembly, Step 1).^41^ With further evaporation of solvent, W species/PEO-*b*-PS/Ag NCs hybrid micelles packed into an ordered mesostructured organic–inorganic nanocomposite, which can be solidified by annealing at 100 °C (Step 2). The as-made composites were finally treated sequentially in nitrogen at 350 °C and in air at 400 °C to remove templates, resulting in ordered mesoporous Ag/WO_3_ composites (Step 3).

**Figure 1:**
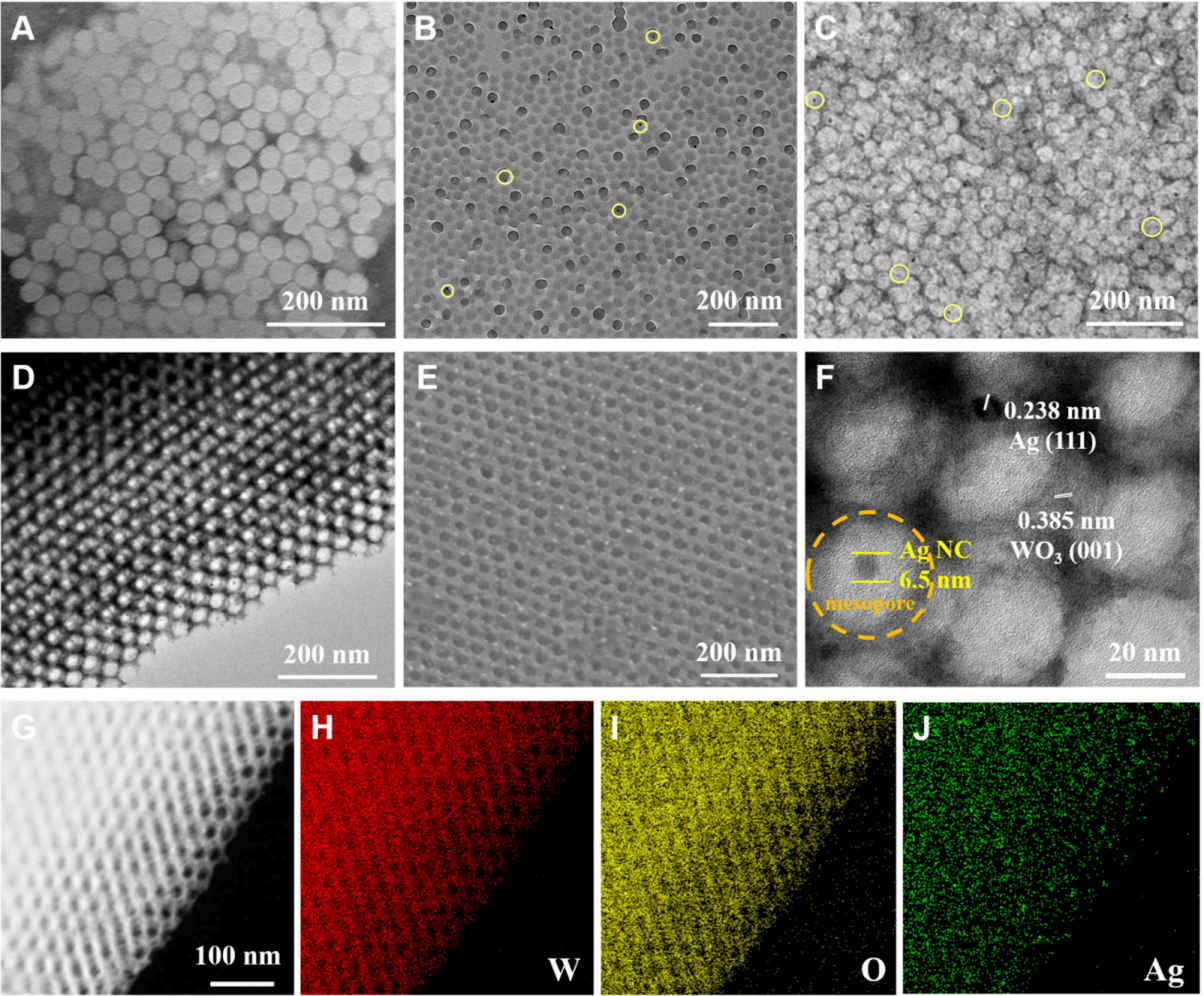
TEM images of W species/PEO-*b*-PS micelles (A), PEO-*b*-PS/Ag NCs micelles (B), W species/PEO-*b*-PS/Ag NCs micelles (C), and ordered mesostructural Ag/WO_3_ nanocomposites (D). (E) FESEM image, (F) HRTEM image, and (G) HAADF-STEM image and the corresponding EDX elemental mapping images (H–J) of W, O, and Ag elements of ordered mesoporous 0.5-Ag/WO_3_ composites. The representative Ag NCs encapsulated in PS cores of micelles are highlighted by bright yellow circles.

Transmission electron microscopy (TEM) was employed to characterize the samples obtained during the co-assembly process. When tungsten species and hydrophobic Ag NCs co-assemble with PEO-*b*-PS copolymers, respectively, hydrophilic tungsten species can interact with PEO segments to form micelle shells (Figure 1A), while Ag NCs are well encapsulated in the PS core of micelles via hydrophobic interaction (Figure 1B). When they assemble with PEO-*b*-PS simultaneously, ordered mesostructured W species/PEO-*b*-PS/Ag NCs hybrid composites can be obtained (Figure 1C). TEM and field emission scanning electron microscopy (FESEM) images indicate that the obtained mesoporous Ag/WO_3_ materials display a typical ordered mesoporous structure with spherical mesopores of approximately 35.0 nm (Figures 1D,E and S2). Highly dispersed Ag NCs confined in the uniform mesopores without aggregation could also be observed clearly from the TEM image, due to the strong interaction between PS segments and surface ligands of NCs that ensured an effective inclusion of NCs in the PS domain during the RSCA procedure. The high-resolution TEM (HRTEM) image of mesoporous Ag/WO_3_ (Figure 1F) reveals that Ag NCs were stably confined in the mesopores, and the size of Ag NCs was measured to be 6.5 nm, which is consistent with the diameter of pre-synthesized Ag NCs. It further confirms that the confinement effect of mesopores can effectively prevent Ag NCs from agglomeration during calcination. From the HRTEM image, the interplanar spacings of Ag NCs and WO_3_ were calculated as 0.238 and 0.385 nm, which can be indexed to (111) of cubic Ag and (001) of orthorhombic WO_3_, respectively. Moreover, selected area electron diffraction (SAED) analysis (Figure S3) shows spotty diffraction rings, indicative of a polycrystalline WO_3_ framework. Furthermore, the high-angle annular dark-field scanning transmission electron microscopy (HAADF-STEM) image (Figure 1G) of mesoporous Ag/WO_3_ also shows an ordered mesoporous structure with uniform mesopores (*ca*. 35.0 nm in diameter) and a wall thickness of 5.0 nm. The corresponding energy dispersive X-ray (EDX) element mappings (Figure 1H–J) and SEM-EDX element mappings (Figure S4) in low magnification indicate the homogeneous distributions of W, O, and Ag elements over large domains, providing additional evidence for the formation of evenly Ag NCs-decorated mesoporous WO_3_ via the unique regiospecific co-assembly.

**Figure 2:**
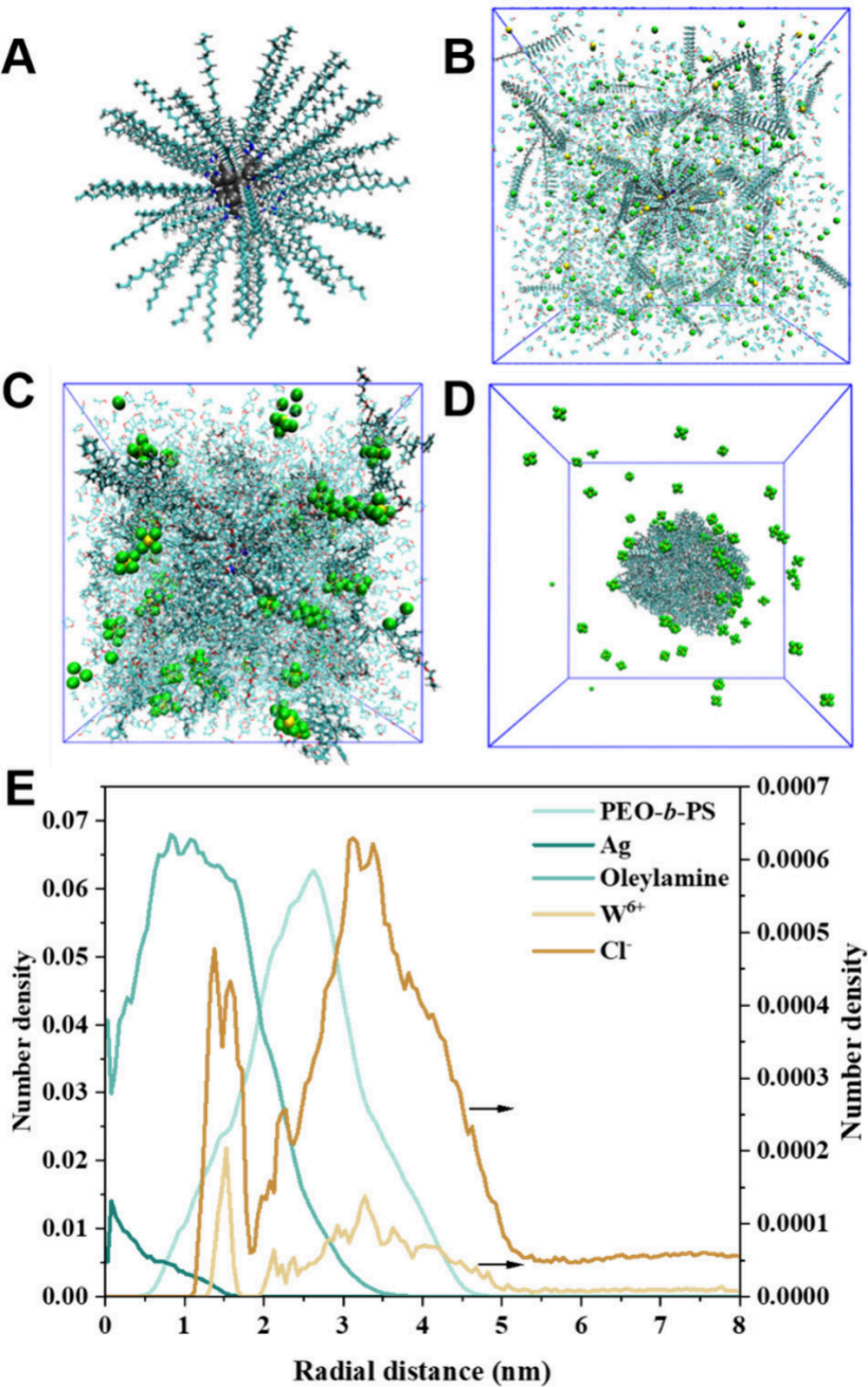
Snapshots of the initial configuration of Ag@OAm (A), the initial simulation system (B), and the simulation system after partial (C) and complete (D) removal of solvent molecules. (E) Radial density profile of molecules in aggregate. Ag, W, Cl atoms and OAm molecules were sketched in van der Waals model. PEO-*b*-PS and solvent molecules were shown in the Licorice model and line model, respectively. The hydrogen, carbon, oxygen, nitrogen, W, Cl, and Ag atoms are marked with white, cyan, red, blue, yellow, green and gray, respectively.

Molecular dynamics (MD) simulations were performed to further investigate the regiospecific co-assembly process. First, an Ag cluster surrounded by oleylamine molecules was built in the simulation box with a size of 16 × 16 × 16 nm^3^ to represent Ag@OAm, as shown in Figure 2A. PEO-*b*-PS, metal oxide precursors (W^6+^ and Cl^–^), and solvent (tetrahydrofuran and ethanol) molecules were randomly introduced into the simulation box as the initial configuration of the simulation system (Figure 2B), and all molecules were allowed to move freely. The initial simulation system can reach equilibrium after 50 ns of simulation, and then a portion of solvent molecules were randomly removed from the system every 10 ns, leading to the gradual gathering of residual molecules in the simulation system (Figure 2C). After the complete removal of solvent molecules, PEO-*b*-PS molecules and precursors co-assembled into composite micelles with Ag@OAm restricted in the core and W species distributed around the shell (Figure 2D), consistent with results from TEM analysis (Figure 1A–C), which was also confirmed by the radial density profile of molecules in aggregates (Figure 2E). The interaction energies analysis reveals that the strong interaction between PS segments and Ag@OAm effectively stabilizes the nanocrystal encapsulated in the core of micelle and leads to regiospecific co-assembly (Figure S5).

**Figure 3:**
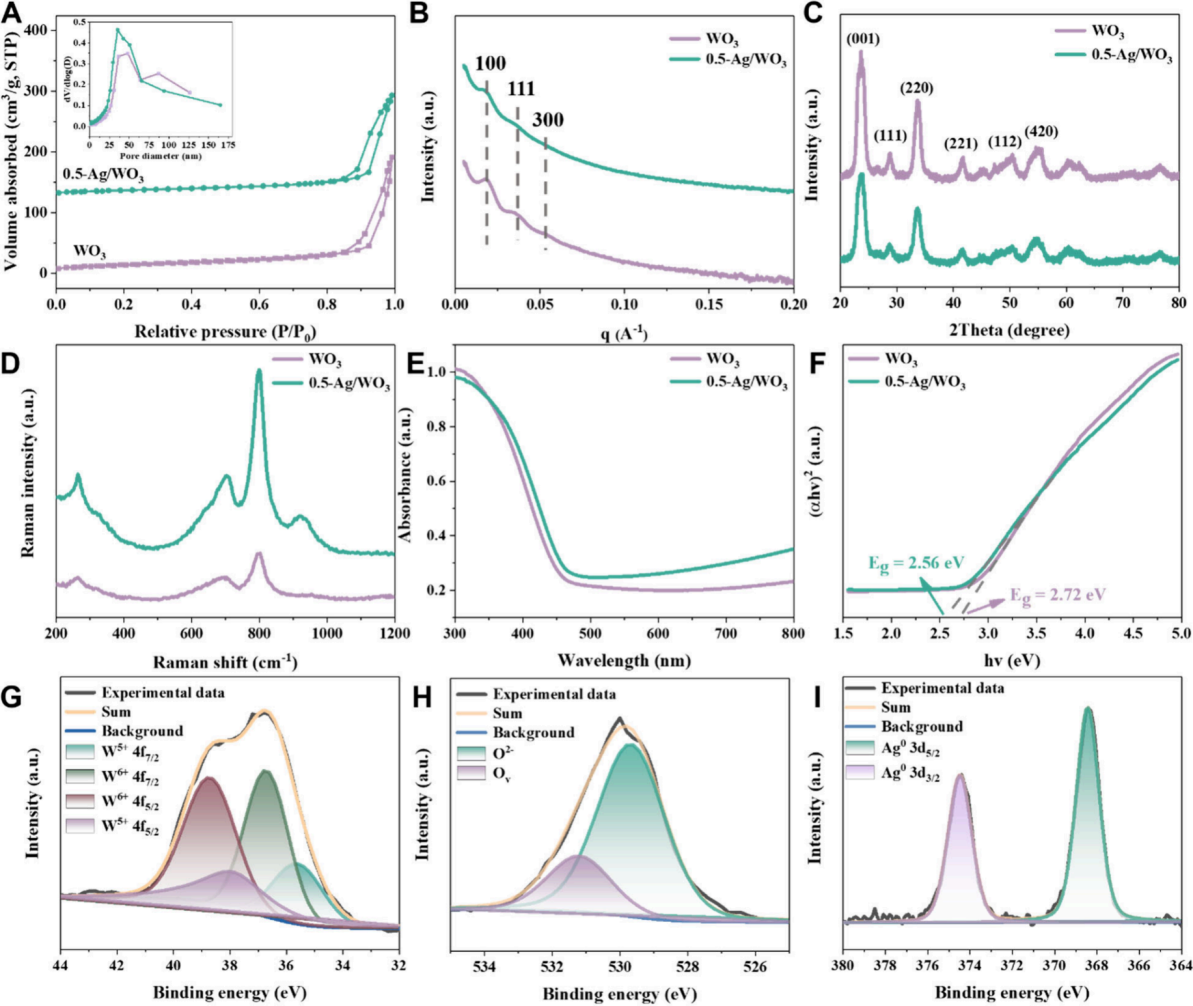
(A) N_2_ adsorption–desorption isotherms and pore size distribution curves (inset), (B) SAXS patterns, (C) XRD patterns, (D) Raman spectra, (E) UV–vis diffuse reflectance spectra, and (F) Tauc plots of mesoporous WO_3_ and 0.5-Ag/WO_3_. XPS spectra of mesoporous 0.5-Ag/WO_3_ in the vicinity of W 4f (G), O 1s (H), and Ag 3d (I). N_2_ adsorption–desorption isotherms of mesoporous 0.5-Ag/WO_3_ are offset vertically by 110 cm^3^ g^–1^.

Nitrogen adsorption–desorption isotherms of mesoporous WO_3_ and 0.5-Ag/WO_3_ samples show typical type-IV curves with an H1-type hysteresis loop (Figure 3A), suggesting the existence of large spherical mesopores. The narrow pore size distribution (Figure 3A inset) derived from the adsorption branch based on the Broekhoff de Boer spherical model demonstrates that both samples have a uniform pore size of about 35.0 nm, consistent with the results based on TEM analysis. The Brunauer–Emmett–Teller specific surface areas of mesoporous WO_3_ and 0.5-Ag/WO_3_ were calculated to be 51.9 and 42.9 m^2^/g, respectively, and total pore volumes of these samples are 0.29 and 0.26 cm^3^g^–1^ (Table S1), respectively. The slight reduction of specific surface area and total pore volume can be attributed to the high dispersion and precise decoration of Ag NCs within the pore wall of the mesoporous WO_3_. Small-angle X-ray scattering (SAXS) patterns of both samples show three strong scattering peaks (Figure 3B), which can be indexed to the (100), (111) and (300) reflections of the ordered face-centered cubic mesostructure with space group of *Fm*3*m*.^4^ It suggests that decoration with Ag NCs via RSCA has a negligible impact on the formation of regular mesostructure of WO_3_.

X-ray diffraction (XRD) measurements (Figure 3C) indicate that mesoporous WO_3_ and 0.5-Ag/WO_3_ samples have similar patterns and well-resolved diffraction peaks assigned to the crystalline orthorhombic phase of WO_3_ (PDF No. 20–1324, *a* = 0.7384, *b* = 0.7512, *c* = 0.3846 nm). Notably, no diffraction peaks from Ag NCs were detected in the 0.5-Ag/WO_3_ samples, and such a phenomenon can be attributed to the high dispersion of ultrasmall Ag NCs and the shielding effect of the crystalline WO_3_ wall. Raman spectra (Figure 3D) of those two samples show three strong scattering peaks at 267, 709, and 800 cm^–1^. The peak at 267 cm^–1^ can be assigned to the O–W^6+^–O bond bending vibration, while bands at 709 and 800 cm^–1^ correspond to the asymmetric and symmetric stretching modes of O–W^6+^–O bond, respectively.^47^ Particularly, the intensity of the three peaks was increased for mesoporous 0.5-Ag/WO_3_, owing to the surface-enhanced Raman scattering effect of Ag NCs.^48^ UV–vis diffuse reflectance spectroscopy (DRS) characterization (Figure 3E) indicates that the absorption intensity of mesoporous 0.5-Ag/WO_3_ in the visible region was slightly higher than that of mesoporous WO_3_, and the band gap of mesoporous 0.5-Ag/WO_3_ was estimated to be 2.56 eV via the Tauc plots (Figure 3F), much lower than that of mesoporous WO_3_ (2.72 eV). These results imply that the existence of rich interfaces of Ag-WO_3_ and Ag NCs donate electrons into the conduction band of WO_3_.^49,50^

The information on the chemical states of W, O, and Ag was investigated by X-ray photoelectron spectroscopy (XPS) analysis. The narrow-scan W 4f XPS spectrum of mesoporous WO_3_ (Figure S6A) shows two peaks at 36.2 and 34.1 eV, attributed to W^6+^ 4f_5/2_ and W^6+^ 4f_7/2_, respectively. By contrast, 0.5-Ag/WO_3_ composite (Figure 3G) displays two doublets at 38.8 and 36.8 eV assigned to W^6+^ 4f_5/2_ and 4f_7/2_ and another two peaks at 38.0 and 35.6 eV attributed to W^5+^ 4f_5/2_ and 4f_7/2_, respectively.^4^ These results indicate that 0.5-Ag/WO_3_ contains both W^6+^ and W^5+^ states, reflecting that more defects were generated or incomplete W–O binding occurred in the 0.5-Ag/WO_3_ sample. On the other hand, the O 1s spectra of mesoporous WO_3_ (Figure S6B) and 0.5-Ag/WO_3_ (Figure 3H) both exhibit two peaks indexed to oxygen vacancies (531.0 eV) and lattice oxygen O^2–^ (530.4 eV), respectively.^51,52^ While the content of oxygen vacancies in mesoporous Ag/WO_3_ was calculated to be 24.7% (Table S1), more than that in mesoporous WO_3_ (21.8%). As to Ag element, the peaks at 374.4 and 368.4 eV can be assigned to Ag^0^ 3d_3/2_ and Ag^0^ 3d_5/2_, respectively, while no peak belonging to Ag^+^ was observed in Figure 3I,^53^ indicating the presence of well-reserved metallic Ag species after calcination at 400 °C. This result agrees well with XRD and Raman spectra.

**Figure 4:**
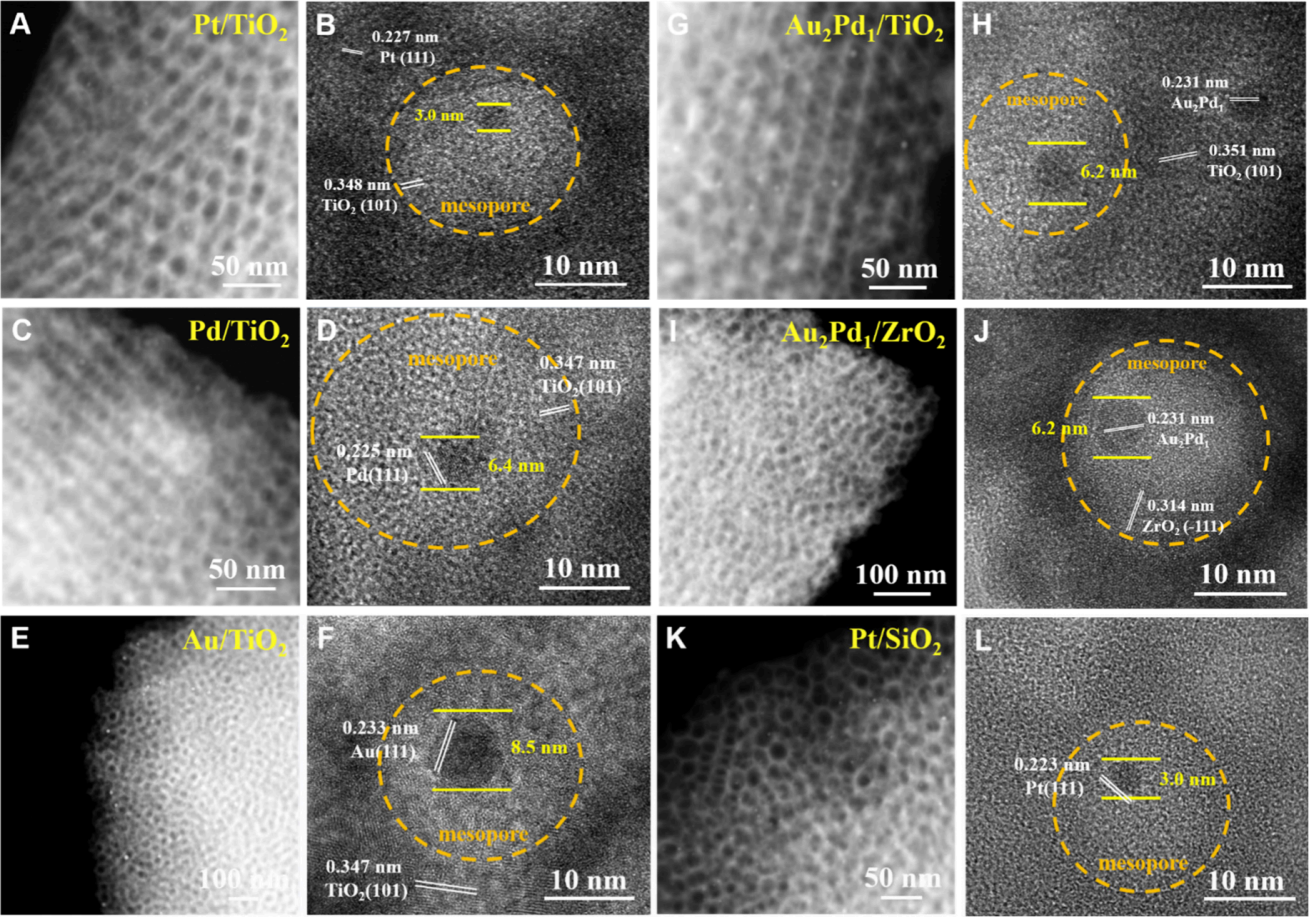
HADDF-STEM (A, C, E, G, I, K) and HRTEM (B, D, F, H, J, L) images of mesoporous Pt/TiO_2_, Pd/TiO_2_, Au/TiO_2_, Au_2_Pd_1_/TiO_2_, Au_2_Pd_1_/ZrO_2_, and Pt/SiO_2_.

To study the generality of the proposed RSCA method (Scheme 1), uniform hydrophobic Pt, Pd, and Au NCs of certain size (3.0–8.5 nm) were pre-synthesized with OAm as the ligand (Figure S7 and Table S2) and used to co-assemble with PEO-*b*-PS and partially hydrolyzed titanium butoxide. The co-assembly process was confirmed by electron microscopy characterization (Figure S8). TEM and HADDF-STEM images of mesoporous Pt/TiO_2_ (Figures S9A and 4A), Pd/TiO_2_ (Figures S9B and 4B), and Au/TiO_2_ (Figures S9C and 4C) show that noble metal NCs are in a high dispersion state in the uniform mesopores. HRTEM images (Figure 4B, D, and F) show that Pt NCs of 3.0 nm, Pd NCs of 6.4 nm, and Au NCs of 8.5 nm were well confined in the uniform mesopores, respectively, and the sizes of these noble metal NCs in mesoporous composites are consistent with that of pre-synthesized metal nanocrystals. The corresponding EDX element mapping images (Figure S9B, D, and F) indicate the homogeneous distribution of Ti, O, and noble metals, further demonstrating the successful synthesis of mesoporous metal NCs/TiO_2_. Moreover, this RSCA strategy is applicable to synthesize functional mesoporous metal oxides with pore wall modified by component-customized alloy nanocrystals. For example, the pre-synthesized hydrophobic Au–Pd alloy NCs with different metal ratios, e.g., Au_2_Pd_1_ (Figure S10A,B) and Au_1_Pd_1_ (Figure S10C,D), can be used in the RSCA method, as confirmed by electron microscopy characterization (Figure S11). After the removal of templates, the obtained mesoporous Au_2_Pd_1_/TiO_2_ and Au_1_Pd_1_/TiO_2_ exhibit a well connected mesoporous structure and alloy NCs confined in mesopores without agglomeration (Figures 4G,H and S12). Furthermore, other framework precursors such as zirconium butoxide and tetraethyl orthosilicate can be used to generate hydrophilic oligomers to co-assemble with PEO-*b*-PS and hydrophobic noble metal NCs via this versatile RSCA method, which gives rise to diverse mesoporous noble metal NCs/oxide composites, including mesoporous Au_2_Pd_1_/ZrO_2_, Au_1_Pd_1_/ZrO_2_, and Pt/SiO_2_ (Figures 4I–L, S13, and S14), further confirming the universality of this method.

### Gas Sensing Studies

Considering the ordered and interconnected mesoporous structure, high surface areas and rich fully exposed noble metal–metal oxide interfaces of the obtained mesoporous Ag/WO_3_ nanocomposites, we investigated their application possibility in chemiresistive sensors toward NO that is a common pollutant gas and relevant to serious environmental problems (e.g., acid rain and photochemical smog) and health state (e.g., emphysema).^54^ First, gas sensing devices were fabricated using mesoporous 0.5-Ag/WO_3_ as the sensing layer on ceramic tube substrates. The assembled device and electric circuit for nitric monoxide gas sensing measurements are shown in Figure S15. To optimize the working temperature of the sensors, the mesoporous 0.5-Ag/WO_3_ sensors were tested toward NO gas with a concentration of 25 ppm at different temperatures, considering the threshold limit value of NO is 25 ppm according to the American Conference of Government Industrial Hygienists.^55^

**Figure 5:**
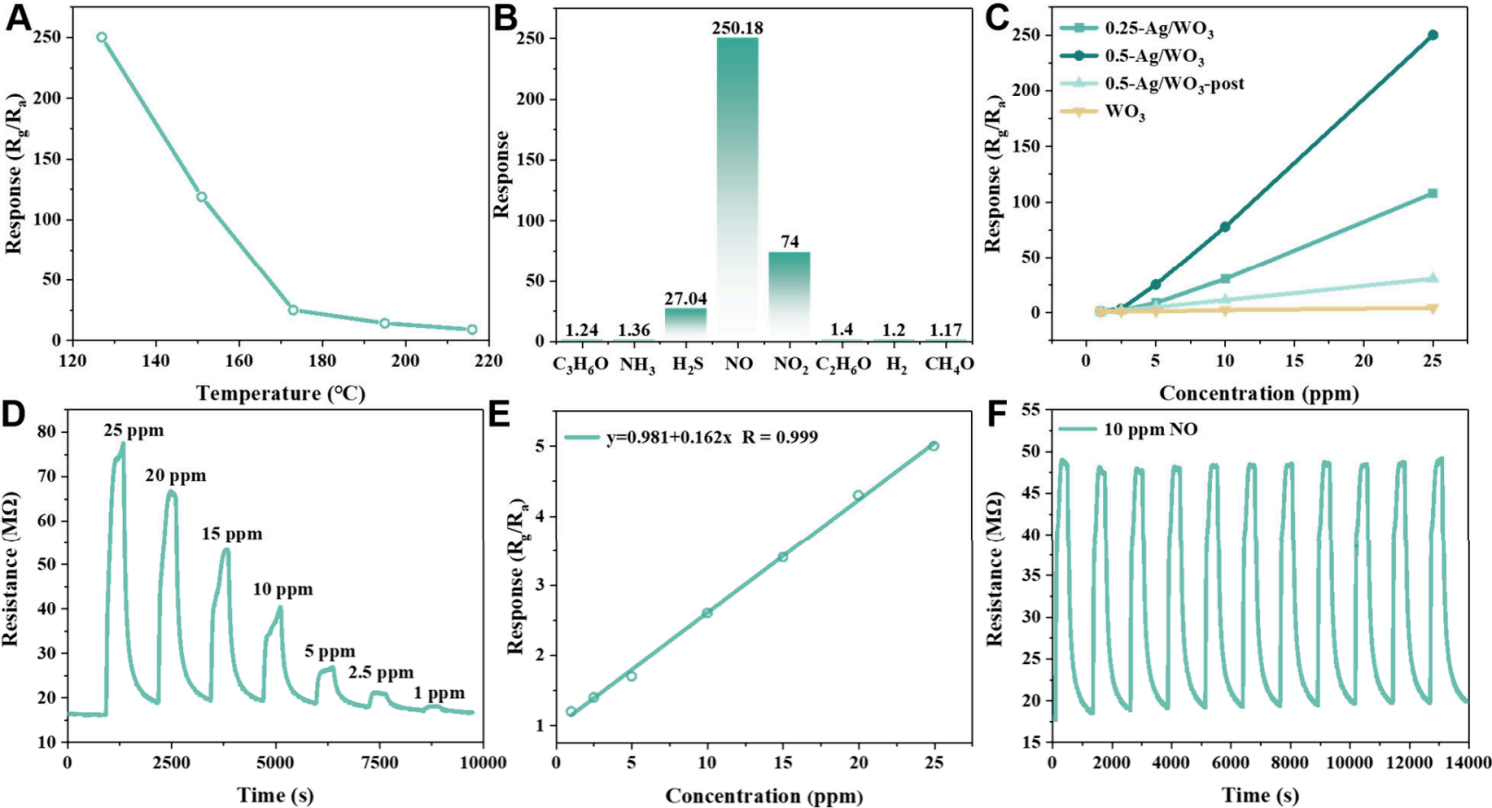
(A) Relationship between working temperatures and responses of a ceramic tube-based mesoporous 0.5-Ag/WO_3_ sensor in 25 ppm of NO. (B) The response of mesoporous 0.5-Ag/WO_3_ toward different gases was 25 ppm at 127 °C. (C) Relationship between NO concentrations and responses of mesoporous WO_3_, 0.25-Ag/WO_3_, 0.5-Ag/WO_3_, and 0.5-Ag/WO_3_-post sensors based on a ceramic tube at 127 °C. (D) Dynamic response curve of mesoporous 0.5-Ag/WO_3_ based MEMS sensor exposed to various concentrations (1–25 ppm) of NO at 127 °C. (E) Relationship between NO concentration and response of the mesoporous 0.5-Ag/WO_3_ based MEMS sensor in a log–log plot. (F) Repeating response and recovery curve of the mesoporous 0.5-Ag/WO_3_ based MEMS sensor toward 10 ppm of NO at 127 °C.

The mesoporous 0.5-Ag/WO_3_ sample was first tested in the range of 120–220 °C (Figure 5A): the response of the sensor decreased as the working temperature increased, and the response to 25 ppm of NO can reach 250 at 127 °C. It indicates that lower temperature is more suitable for sensitive NO detection. Moreover, considering the too long response/recovery time (>10 min) of the sensor when the working temperature is below 127 °C, the optimal working temperature was set at 127 °C for further study.

Mesoporous 0.5-Ag/WO_3_ was then tested in 25 ppm of different gases at 127 °C for selectivity appraisal (Figure 5B). The mesoporous 0.5-Ag/WO_3_ based sensor shows a high response of about 250 toward 25 ppm of NO, three times higher than that to NO_2_, let alone low responses of about 1 to interference gases (e.g., ethanol, acetone, and ammonia) of 25 ppm, suggesting a superior sensing selectivity to NO. For comparison, mesoporous WO_3_ and 0.25-Ag/WO_3_ (Ag content: 0.25 wt.%) were synthesized following a similar strategy by adjusting the loading amount of Ag NCs (Figures S16 and 17), and 0.5-Ag/WO_3_-post was synthesized through conventional impregnation-reduction method (Figure S18). The sensing performances of the obtained materials were evaluated toward NO with a concentration range from 1 to 25 ppm at 127 °C (Figure 5C). The sensitivity of the materials follows the order of mesoporous WO_3_ < 0.25-Ag/WO_3_ < 0.5-Ag/WO_3_, consistent with that of the Ag NCs content, confirming that construction of Ag-WO_3_ interfaces can significantly improve the NO sensitivity. Nevertheless, 0.5-Ag/WO_3_-post with higher Ag content showed lower NO sensitivity than mesoporous 0.25-Ag/WO_3_, owing to the large diameter and uncontrollable distribution of Ag nanoparticles formed via the traditional postloading method. The better sensing properties of mesoporous Ag/WO_3_ can be attributed to the precise decoration of size-customized Ag NCs in the pore wall with well-retained mesoporous WO_3_ via an effective RSCA strategy. Furthermore, the reversible cycles of the response curve of mesoporous 0.5-Ag/WO_3_ shows retained response of about 78 to 10 ppm of NO (Figure S19), reflecting a reliable gas sensing process. The response and recovery time of mesoporous 0.5-Ag/WO_3_ sensor to 10 ppm of NO was calculated to be 243 and 171 s from the dynamic response-recovery characteristic curve (Figure S20). Such response-recovery dynamics of the mesoporous Ag/WO_3_-based sensor can be assigned to the presence of silver nanocrystals and the interconnected mesoporous structure for the gas diffusion and gas-catalytic sites interaction. Besides, the long-term stability of the 0.5-Ag/WO_3_ based sensor was studied and is shown in Figure S21. The sensor was exposed to 10 ppm of NO at 127 °C for a sensing test every 5 days and showed responses with relative deviations of 2% over nearly two months, implying the good long-term sensing stability of the mesoporous 0.5-Ag/WO_3_-based sensor toward NO. The good sensing stability can be ascribed to the well-retained ordered mesoporous structure and highly dispersed Ag NCs of mesoporous 0.5-Ag/WO_3_ even after two-month sensing stability tests (Figure S22). Additionly, the anti-humidity ability of mesoporous 0.5-Ag/WO_3_ was investigated (Figure S23), and the response toward 10 ppm of NO at 127 °C declined by 28% as the ambient humidity increased from 44% to 97%. Such a result is mainly due to plenty of water molecules occupying active sites, hindering the sensing process of NO over the mesoporous 0.5-Ag/WO_3_. Compared to the WO_3_-based NO sensors reported before (Figure S24), our gas sensor based on mesoporous 0.5-Ag/WO_3_ exhibits better comprehensive performance due to its high porosity, abundant oxygen vacancies, and rich Ag-WO_3_ interfaces.

Notably, in order to evaluate the practical application of the mesoporous Ag/WO_3_ based sensors for NO sensing detection, the sensing materials were deposited on the electrodes of the micro electromechanical system (MEMS) chips with high integration and low energy consumption (Figure S25). The dynamic response of the mesoporous 0.5-Ag/WO_3_ based MEMS sensor toward various concentrations (1–25 ppm) of NO was also measured at 127 °C, and the result is displayed in Figure 5D. The response to 25 ppm of NO is about 5.0. The lower response compared to that of ceramic tube-based sensors can be attributed to much less usage of sensing materials and the unique device features of MEMS chips and the dynamic testing system. Nevertheless, the MEMS sensor still has a response of 1.2 to 1 ppm of NO, and exhibits an excellent linearity relationship in log–log plot between the response values and NO concentrations with a correlation coefficient of 0.999 (Figure 5E). The reversible response cycles of the MEMS sensor show retained response of about 2.6 toward 10 ppm of NO even after 10 cycles of measurements (Figure 5F), reflecting a good repeatability and durability of this MEMS sensor. Such an excellent sensing performance of the mesoporous 0.5-Ag/WO_3_ based MEMS sensing device makes it a potential candidate for quantitative detection of NO in practical applications.

### NO Sensing Mechanism

**Figure 6:**
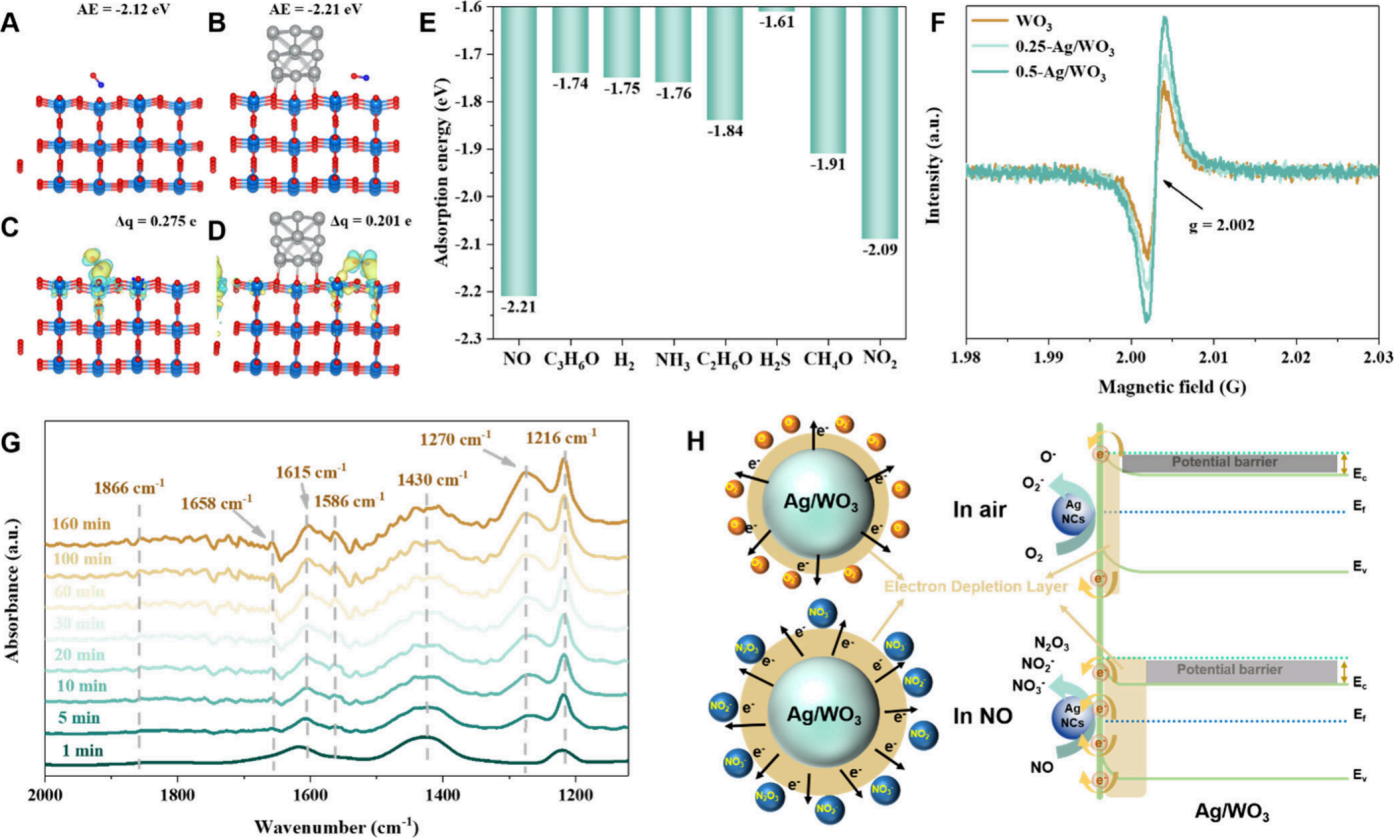
Optimized structure and corresponding adsorption energy (AE) of NO molecule on mesoporous (A) WO_3_ and (B) Ag/WO_3_. Charge density difference of (C) mesoporous WO_3_ and (D) Ag/WO_3_ with adsorption of NO, respectively. (E) Statistical chart of adsorption energy of various gases on mesoporous Ag/WO_3_. (F) EPR spectra of ordered mesoporous WO_3_, 0.25-Ag/WO_3_ and 0.5-Ag/WO_3_ materials. The symmetrical EPR signal at g = 2.002 is attributed to the unpaired electrons in the oxygen vacancies. (G) *In situ* time-resolved DRIFTS spectra of NO_*x*_ species chemisorbed on mesoporous 0.5-Ag/WO_3_ recorded at 1100–2000 cm^–1^ upon exposure to an O_2_ atmosphere at 127 °C as a function of reaction time. (H) Schematic diagram of energy band structure and electron-transfer process for ordered mesoporous Ag/WO_3_ sensitive materials exposed in air and NO/air mixture at 127 °C.

Since the gas sensing process of semiconducting metal oxides involves gas–solid interactions (e.g., adsorption, desorption, and conversion), DFT calculations were carried out to study gas adsorption energies on the surface of WO_3_ and Ag/WO_3_, aiming to gain underlying information about the excellent selectivity of the sensors. As shown in Figure 6A and B, NO molecules can be absorbed over mesoporous WO_3_ and mesoporous Ag/WO_3_ with adsorption energy of −2.12 and −2.21 eV, respectively, implying that the modification with Ag NCs is beneficial to the adsorption of NO molecules over mesoporous WO_3_. Notably, as for the charge density difference of the NO molecule adsorbed on mesoporous Ag/WO_3_, charge transfer can occur between both N and O atoms of NO molecules and W atoms of the materials, and this is different from the case of the NO molecule adsorbed on WO_3_, in which charge transfer only occurs between N and W atoms (Figure 6C,D). This may explain how the functionalization of mesoporous WO_3_ by Ag nanoparticles is beneficial for NO stabilization on WO_3_, thus enhancing the chemisorption of NO molecules. The adsorption energy of other gases (Figures 6E and S26) on mesoporous Ag/WO_3_ was calculated to be less negative than that of NO, suggesting that the NO molecule is much easier to be chemsorbed on mesoporous Ag/WO_3_, accounting for the superior sensing selectivity to NO. Moreover, Ag is a well-known catalyst that can reduce the activation energy of NO oxidization,^56,57^ and the modification with Ag NCs on WO_3_ can endow materials with more oxygen vacanccies, which was demonstrated by XPS and EPR characterization results. (Figure 6F and Table S1). Therefore, mesoporous Ag/WO_3_ materials with abundant Ag-WO_3_ interfaces as active sites for NO oxidation display higher NO sensitivity.

To gain more information about the gas sensing process and elucidate the NO sensing mechanism of the mesoporous Ag/WO_3_, *in situ* DRIFTS was applied to characterize the reaction intermediates and products during heating the sensing materials in the presence of the NO/air mixture. The obtained *in situ* time-resolved DRIFTS spectra are shown in Figure 6G, and the absorption bands and corresponding assignments are summarized in Table S4. The absorption peak at around 1216 cm^–1^ corresponds to the NO_2_^–^ species, and the peaks at 1586 and 1866 cm^–1^ are attributed to N_2_O_3_. Besides, NO_3_^–^ species can be well distinguished from absorption bands of different vibration modes at about 1270, 1430, 1615, and 1658 cm^–1^.^58,59^ The intensity of all absorption bands became pronounced as reaction time passed by, while no peak assigned to physisorbed NO_2_ was observed, mainly due to rapid conversion of NO to chemisorbed NO_*x*_ species in the presence of highly dispersed Ag NCs. According to the *in situ* DRIFTS results, the reactions between NO gas molecules and the abundant oxygen species of mesoporous Ag/WO_3_ during the sensing process can be summarized as follows:
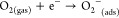
1
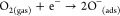
2
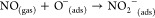
3
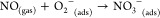
4
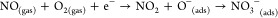
5
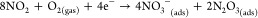
6

As WO_3_ is a typical n-type semiconductor, the adsorbed oxygen molecules can catch free electrons from WO_3_ in the sensing layer to form active oxygen species at temperature lower than 150 °C (mainly O_2_^–^ and O^–^, eqs 1 and 2),^60,61^ leading to the increase of resistance. Upon exposure in a NO atmosphere, NO can be oxidized by active oxygen species in the sensing layer to produce NO_2_^–^, N_2_O_3_, and NO_3_^–^ species (eqs 3–6), and free electrons as carriers in the sensing layer can be further taken away during this redox reaction process. This can cause the reduction of electron concentration and the increase of electron depletion layer (EDL) thickness and potential barrier between the neighboring grains, and thus the further increase of resistance of the sensing materials (Figure 6H).^62^ After a period of equilibrium, mesoporous Ag/WO_3_ was purged with clean air to remove NO_*x*_ species and re-exposed to fresh air, making the resistance return to the original level (Figure S27). When NO sensing over mesoporous Ag/WO_3_ is conducted at temperature higher than 150 °C, active oxygen species are dominated by O^2–^ and O^–^ (eqs S1, S2),^60,63^ resulting in thick EDL and high resistance of material in an air atmosphere. Upon exposure in NO atmosphere, NO can be oxidized easily to chemisorbed NO_*x*_ species (eqs S3–S5), while little electron was taken away from sensing material, inducing slight raise of EDL thickness and resistance, namely, lower sensing response (Figure S28), which accounts for low temperatures (below 150 °C) being beneficial for NO sensing. Overall, the improved NO sensing performance can be ascribed to two main favorable factors achieved via RSCA: (I) The decoration of customized Ag NCs of 6.5 nm, a good NO oxidization catalyst, because it can not only enhance the chemisorption of NO on WO_3_, but also endow the sensing materials with more oxygen vacancies. (II) The well-defined interconnected mesoporous structure, which offers mesoporous channels for Knudsen diffusion of NO molecules and effectively prevents Ag NCs from sintering to maintain abundant Ag-WO_3_ interfaces as sensing active sites, resulting in stable NO sensitivity.

## Conclusion

In summary, an efficient regiospecific co-assembly (RSCA) method was developed to facilely and generally synthesize customized noble metal nanocrystal-functionalized mesoporous metal oxides with large mesopores of 35.0 nm, a diverse crystalline framework (e.g., WO_3_, TiO_2_, and ZrO_2_), and highly dispersed noble metal NCs (e.g., Ag, Pt, Au, Pd, and their nanoalloys) precisely deposited in the interconnected mesopores. As a typical example, mesoporous 0.5-Ag/WO_3_ nanocomposites synthesized via this RSCA method have uniform Ag nanoparticles highly distributed in the mesoporous WO_3_ matrixes, endowing materials with rich catalytic active sites (Ag-WO_3_ interfaces), huge amount of active oxygen vacancies, and more favorable for chemisorption of NO molecules. As a result, gas sensors based on mesoporous 0.5-Ag/WO_3_ exhibit excellent gas sensing performance with a response of 250 toward 25 ppm of NO and high selectivity toward NO at 127 °C. In addition, the sensors show good long-term stability and reliability in response to trace NO with excellent linearity relationship in log–log plot between the response values and NO concentrations, enabling a real-time detection of NO concentration. The NO sensing mechanism study based on DFT calculation, EPR and *in situ* DRIFTS analysis disclosed a significant change in the physical EDL thickness of the mesoporous Ag/WO_3_ sensing layer, along with the enhanced selective chemisorption and conversion of NO molecules into chemisorbed NO_2_^–^, N_2_O_3_ and NO_3_^–^ species on the WO_3_ framework with abundant active oxygen species. This RSCA method provides a great inspiration for rational design and universal synthesis of nanostructural NMs/MOs composites which can find wide applications in various fields, such as heterogeneous catalysis, chemical sensing, energy storage, and biological detection.

## Supporting Information Available

Experimental details about materials synthesis and performance evaluation; structural characterizations and gas sensing performance evaluation of materials tests, experimental details of gas sensing measurement; details about molecular dynamics simulations and density functional theory calculations; and Tables S1–S4 (PDF)
