# Supplementary material for: A Regiospecific Co-Assembly Method to Functionalize Ordered Mesoporous Metal Oxides with Customizable Noble Metal Nanocrystals
Source: ACS Cent Sci. 2024 Nov 21;10(12):2274–84. doi: 10.1021/acscentsci.4c01592 (PMC11672546; doi:10.1021/acscentsci.4c01592)
Supplement: Supplementary file 1 — oc4c01592_si_001.pdf [file oc4c01592_si_001.pdf]

Supporting Information

A Regiospecific Co-assembly Method to Functionalize Ordered Mesoporous Metal Oxides with Customizable Noble Metal Nanocrystals

Jichun Li[†,ψ], Lingxiao Xue[†], Yu Deng[⊥], Xiaowei Cheng[†], Junhao Ma[‡,*], Wenhe Xie[†,*], Meihua Chen[†,*], and Yonghui Deng[†,ψ,*]

[†]Department of Chemistry, Shanghai Stomatological Hospital & School of Stomatology, State Key Laboratory of Molecular Engineering of Polymers, Shanghai Key Laboratory of Molecular Catalysis and Innovative Materials, Fudan University, Shanghai 200433, P. R. China

E-mail: yhdeng@fudan.edu.cn (Yonghui Deng), chenmeihua@fudan.edu.cn (Meihua Chen) whxie@fuadn.edu.cn (Wenhe Xie)

[⊥]State Key Laboratory for Modification of Chemical Fibers and Polymer Materials, College of Materials Science and Engineering, Donghua University, Shanghai 201620, P. R. China

[‡]School of Materials Science and Engineering, Nanyang Technological University, Singapore, 639798, Singapore

E-mail: junhao.ma@ntu.edu.sg (Junhao Ma)

[ψ]State Key Lab of Transducer Technology, Shanghai Institute of Microsystem and Information Technology, Chinese Academy of Sciences, Shanghai 200050, P. R. China

E-mail: yhdeng@fudan.edu.cn (Yonghui Deng)

EXPERIMENTAL SECTION

Chemicals: Monomethyl poly(ethylene oxide) (M_w: 5000 g/mol) was purchased from Aldrich. N,-N,-N',-N",-N"-Pentamethyl di-ethylenetriamine (PMDETA) was purchased from Acros Corp. Tungsten(VI) hexachloride (WCl_6), platinum(II) acetylacetonate ($Pt(acac)_2$), palladium acetylacetonate ($Pd(acac)_2$), gold chloride trihydrate ($HAuCl_4 \cdot 3H_2O$), zirconium butoxide solution (80%), borane-*tert*-butylamine complex and oleylamine (OAm) were purchased from Aladdin Corp. Other chemicals including tetrahydrofuran (THF), ethanol, silver nitrate ($AgNO_3$), tetraethyl orthosilicate (TEOS), titanium butoxide (TBOT), ascorbic acid (AA), cyclohexane, toluene, pyridine, styrene, acetylacetone (AcAc), ethylether, cuprous bromide (CuBr), hydrochloric acid (HCl, 37%), acetic acid (HAc), petroleum ether (b.p. 30 - 60 °C) were purchased from Sino-Pharm Chemical Reagent Co., Ltd.

Synthesis of hydrophobic noble metal/alloy nanocrystals: All the hydrophobic noble metal nanocrystals were synthesized with OAm as ligand. Monodispersed hydrophobic Ag NCs of 6.5 nm in diameter were synthesized by reducing silver nitrate with ascorbic acid as a weak reductant at 25 °C. Typically, 85 mg of $AgNO_3$ and 4 g of OAm were dissolved in 5 mL of toluene under ultrasonication. 175 mg of

AA was then added into the mixed solution under magnetic stirring. After further stirring for 1.5 h in dark, the resultant Ag NCs stabilized by OAm were collected by precipitation with ethanol and centrifugation. The product was washed with ethanol and then redispersed in 40 mL of cyclohexane for further use. The concentration of Ag NCs dispersed in cyclohexane is 110 μg/mL, as determined by inductively coupled plasma optical emission spectrometer (ICP-OES) analysis.

Pt NCs of 3.0 nm, Pd NCs of 6.4 nm and Au NCs of 8.5 nm were synthesized with borane-*tert*-butylamine complex or OAm as reductant according to previous reports.[1-3] Au_2Pd_1 NCs of 6.2 nm were synthesized with OAm as reductant referring to previous report,[3] and Au_1Pd_1 NCs of 6.3 nm was obtained via the same method by simply altering the ratio of $HAuCl_4 \cdot 3H_2O$ and $Pd(acac)_2$. All the obtained hydrophobic nanocrystals were dispersed in cyclohexane, and concentrations were determined by ICP-OES analysis and listed in **Table S2**.

Synthesis of ordered mesoporous 0.5-Ag/WO_3 with pore wall decorated by Ag NCs: The structure-directing agent PEO_{114}-*b*-PS_{274} with a molecular weight of 33600 g/mol was synthesized via atom transfer radical polymerization according to previous reports.[4] Typically, 75 mg of PEO-*b*-PS was dissolved in 5 mL THF by ultrasonic to form solution A, and 300 mg of WCl_6 was dissolved in a mixture of 1 mL of ethanol and 300 μL of acetylacetone to form solution B under stirring of 5 min. Afterwards, solution A and B were mixed under stirring for 0.5 h to form deep green transparent solution, followed by introduction of 125 μL of Ag NCs dispersion. After further stirring for 0.5 h, the resultant mixture was poured into Petri dishes to evaporate solvent in a hood at 25 °C for 24 h, the ambient relative humidity was controlled at 30 - 40%. The as-formed composites on the glass substrate of Petri dishes were further heated at 40 °C in an oven for 12 h to completely remove solvent and annealed at 100 °C for another 12 h to form dark bule transparent film. The film was scraped off and crushed into powder which was thermally treated at 350 °C for 2 h with a ramp of 1 °C/min and then at 500 °C (5 °C/min) for 1 h under nitrogen atmosphere. The obtained product was finally calcined in air at 400 °C for 1h with a

ramp of 2 °C/min to produce mesoporous 0.5-Ag/WO_3. For comparison, ordered mesoporous WO_3 without loading Ag and mesoporous 0.25-Ag/WO_3 were also synthesized through the above method. 0.5-Ag/WO_3-post was synthesized by traditional impregnation-reduction method.[5]

Synthesis of mesoporous TiO_2 with pore wall decorated by noble metal NCs: 20 mg of PEO_{114}-*b*-PS_{274} was dissolved in 5 mL of THF with 40 μL of HCl and HAc, 100 μL of TBOT was then added to the solution. After 1.5-hour stir, 30 - 75 μL of noble metal NCs dispersion was introduced and the mixture was then stirred for 0.5 h. The solvent evaporation, aging and thermal treatment procedure were the same as those for the synthesis of mesoporous Ag/WO_3.

Synthesis of mesoporous ZrO_2 with pore wall decorated by noble metal alloy NCs: 20 mg of PEO_{114}-*b*-PS_{274} was dissolved in 5 mL of THF with 40 μL of HCl and HAc, 134 μL of zirconium butoxide solution was then added to the solution. After 1.5-hour stir, 40 μL of noble metal alloy NCs dispersion was introduced and the mixture was then stirred for 0.5 h. The solvent evaporation, aging and thermal treatment procedure were the same as those for the synthesis of mesoporous Ag/WO_3.

Synthesis of mesoporous SiO_2 with pore wall decorated by Pt NCs: 20 mg of PEO_{114}-*b*-PS_{274} was dissolved in 5 mL of THF with 40 μL of HCl and HAc, 65 μL of TEOS was then added to the solution. After 1.5-hour stir, 75 μL of Pt NCs dispersion was introduced and the mixture was then stirred for 0.5 h. The solvent evaporation, aging and thermal treatment procedure were the same as those for the synthesis of mesoporous Ag/WO_3.

Measurement and characterization: Small-angle X-ray scattering (SAXS) measurements were carried out on Nanostar U small angle X-ray scattering system (Bruker, Germany) with Cu Kα radiation (40 kV, 40 mA). X-ray power diffraction (XRD) patterns were collected on D4 X-ray diffractometer (Bruker, Germany) equipped with Ni-filtered Cu *K*α radiation (40 kV, 40 mA). Transmission electron microscope (TEM) images were taken on HT7800 microscope (Hitachi, Japan). Scanning electron microscope (SEM) images were recorded on S4800 FESEM

(Hitachi, Japan). High-resolution transmission electron microscope (HRTEM) images were taken on field emission transmission electron microscope (FEI, America). Nitrogen adsorption-desorption isotherms were measured at 77 K on a Tristar 3020 analyzer (Micromeritics, USA). Before measurement, the samples were degassed in vacuum at 180 °C for 6 h. The specific surface areas of the samples were calculated through the Brunauer-Emmett-Teller (BET) method. Total pore volumes (V_{total}) were calculated from the adsorbed amount of nitrogen at P/P_0 = 0.995 and pore size distribution curves were obtained from the adsorption branch of isotherms by using the Barrett-Joyner-Halenda (BJH) model. The electron paramagnetic resonance (EPR) data were collected

by A300 (Bruker, Germany). X-ray photoelectron spectroscopy (XPS) analysis was conducted on RBD 147 upgraded PHI 5000C ESCA system (ULVAC-PHI, Japan) using a dual X-ray source. ICP-OES analysis was conducted on iCAP 7400 ICP-OES (Thermo Fisher, USA). Ultraviolet-visible diffuse reflectance spectra (UV-vis DRS) were collected by a Lambd650 (Perkin Elmer, USA). The *in-situ* diffuse reflectance infrared Fourier transform (*in-situ* DRIFT) spectroscopy analysis were performed on Nicolet 6700 Fourier infrared spectrometer (Thermo Fisher, USA) equipped with an *in-situ* heated reaction cell and diffuse reflectance accessory. Each spectrum was recorded using a 64-scan quick sweep mode with a resolution of 4 cm^{-1}. Prior to measurement, the 0.5-Ag/WO_3 sample was heated in the reaction cell at 127 °C with flowing high-pure air (20 mL/min) for 2 h. The background spectrum was recorded in flowing high-pure air (20 mL/min) before NO was purged and subsequently subtracted from the sample spectrum at 127 °C. NO gas and high-pure air were both purged onto the sample surface at a flowing rate of 20 mL/min throughout the testing process and *in situ* DRIFT spectra were recorded at variable times.

Molecular Dynamics (MD) simulation of regiospecific co-assembly: The optimization of the geometry of related molecules was performed via gaussian 16 package at B3LYP/def2svp level.[6] The single point energy was then calculated at B3LYP/def2TZVP level. The acpepy script was employed to obtain the GAFF force

field topology file of them.[7-8] Molecular dynamics simulations were performed using the GROMACS software package (version 2022.6).[9] The total potential energy was given as a combination of valence terms, including bond stretching, angle bending, torsion and nonbonded interactions. The nonbonded interactions between atoms were described by the Lennard-Jones potential, and the standard geometric mean combination rules were used for the van der Waals interactions between different atom species.

In simulation, PEO_{114}-*b*-PS_{274} molecules were simplified to be PEO_4-*b*-PS_{10} with similar phase ratio to avoid consuming computer resources, and each of the systems was initialized by minimizing the energies of the initial configurations using steepest descent method. Following the minimization, a 50 ns MD simulation under NPT ensemble was carried out to equilibrate the system, with a time step of 2 fs. The temperature was annealed linearly from 0 to 298 K within 200 ps, then kept at 298 K till the end of the simulation. In all simulations, the position of Ag atoms and N of oleylamine molecules was restrained with a harmonic potential of 100 $kJ/mol\cdot nm^2$. The temperature was kept constant at 298 K by the v-rescale thermostat algorithm.[10] The pressure was kept constant at 1 atm by the parrinello-rahman algorithm.[11] Bond lengths were constrained using the LINCS algorithm and periodic boundary conditions were applied in all directions.[12] Short-range nonbonded interactions were cut off at 1.0 nm, with long-range electrostatics calculated using the particle mesh Ewald method.[13] Trajectories were stored every 2 ps and visualized using VMD 1.9.3.[14]

Gas sensing performance evaluation based on ceramic tube sensor: The ceramic tube sensor-based gas sensing measurements were performed on a MA1.0 gas sensing measuring system (Narui, China), and the gas sensor devices were fabricated by wet coating and subsequent annealing. The as-synthesized samples were ground into ultrafine powder and dispersed in deionized water to form a thick paste, which was then coated on alumina ceramic tubes equipped with Au electrodes, followed by heating in oven at 50 °C for 12 h to strengthen the interface binding of samples on the

alumina ceramic tubes. To ensure the stability of the devices, the sensors were annealed at 100 °C for 48 h. The stationary state gas distribution method was employed during the testing process, and the target gas sensor was connected in series with a load resistor (R_L). In the sensing test, the tested gases were injected into a test chamber and diluted with air, and the resistance of the sensor in air (R_a) or tested gas (R_g) was measured. The gas response of the sensor is defined as S = R_a/R_g for reducing gases or S = R_g/R_a for oxidizing gases. The response time (t_{res}) is defined as the time taken for R_a changing to R_a + 90% × (R_g − R_a) under the target gas environment, while the recovery time (t_{rec}) is defined as the time taken for R_g changing to R_g − 90% × (R_g − R_a) in the air environment.

Gas sensing performance evaluation based on MEMS sensor: The MEMS sensor-based gas sensing performance was evaluated using a JF02F gas sensing measurement system consisting of two mass flow control modules, a sealed gas chamber with an 8-channel signal acquisition module, and a real-time display. The target gases were diluted to certain concentrations with highly pure air. The MEMS based gas sensor device is composed of a MEMS chip (1 × 1 mm^2), a pair of Au heating electrodes and Au interdigitated electrodes for assessing the resistance of sensing materials. The as-synthesized sample was ground into ultrafine powder, dispersed in ethanol and then drop-coated on MEMS chip. The sensors were annealed at 100 °C for 48 h before test.

Density Function Theory (DFT) calculation: All calculations were finished using first principles based on DFT and were carried out in the Vienna Ab initio Simulation Package code (VASP 5.4.4).[15-16] The generalized gradient approximation (GGA) functional with Perdew-Burke-Ernzerhof (PBE) method was carried out in this work.[17-18] The k-point set of (3 × 2 × 1) was applied for Brillouin zone integration. The cutoff energy was set to 400 eV, and atom coordinates were optimized until the forces were below 0.05 eV·$Å^{-1}$. The self-consistent field iterations were considered converged only if the energy difference was less than 10^{-6} eV. The p(3 × 2)-WO_3 (001) surface layer containing 35 W atoms and 96 O atoms was utilized to construct

the initial model with cell parameters of a = 11.65 Å, b = 15.47 Å, and c = 22.70 Å. Ag_{14} exposed (111) facet was utilized to construct the initial Ag-WO_3 model. A vacuum layer of 15 Å was built in *z* direction to prevent the spurious interlayer interactions. For all models, the bottom two layers were fixed, and the other atoms were allowed to relax. Finally, the adsorption energies (E_{ads}) were calculated as $E_{ads} = E_{ad}/_{sub} - E_{ad} - E_{sub}$, where $E_{ad}/_{sub}$, E_{ad}, and E_{sub} are the total energies of the optimized adsorbate/substrate system, the adsorbate in the structure, and the clean substrate, respectively.

SUPPORTING RESULTS

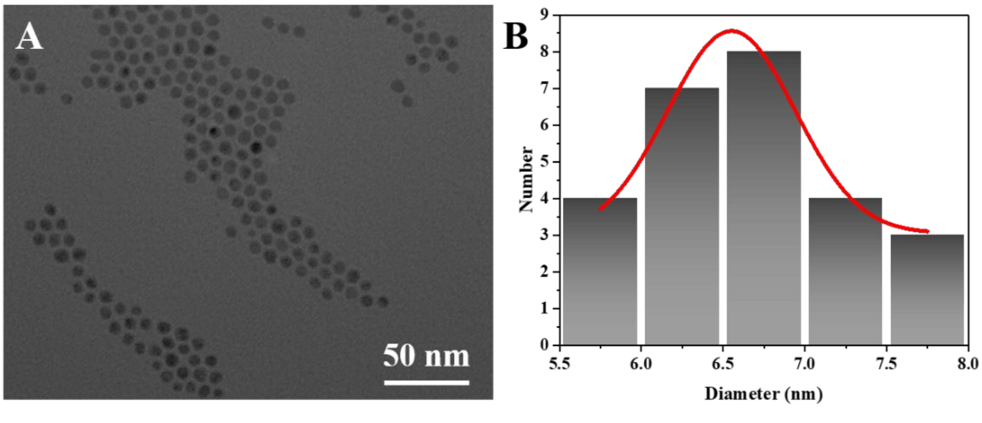

Figure S1. (A) TEM image and (B) the particle size distribution profile of Ag NCs, which indicate a mean diameter of 6.5 nm of the as-synthesized Ag NCs.

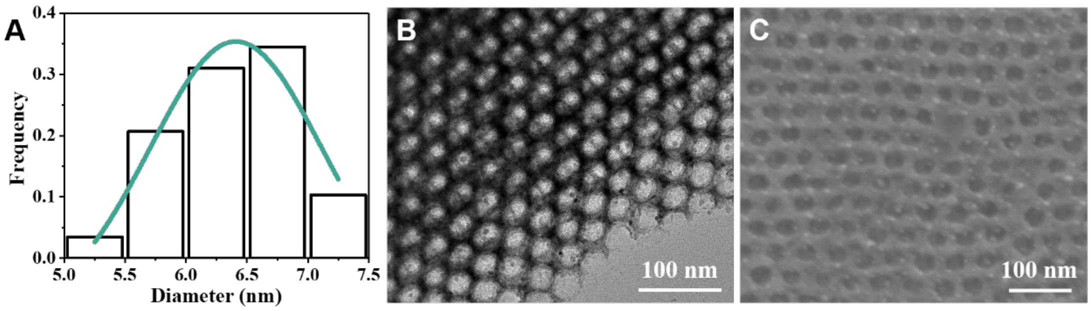

Figure S2. (A) The particle size distribution profile of Ag NCs in mesoporous 0.5-Ag/WO_3 (results calculated from **Figure 2D**). (B)TEM and (C) SEM image of mesoporous 0.5-Ag/WO_3.

The size distribution profile of Ag NCs in mesoporous 0.5-Ag/WO_3 (**Figure S2A)** shows an average size of 6.5 nm, consistent with that of the pre-synthesized Ag NCs. Both TEM and SEM image (**Figure S2B-C)** show that mesoporous 0.5-Ag/WO_3 possess ordered mesoporous WO_3 skeleton with highly dispered Ag NCs well confined in the mesopores.

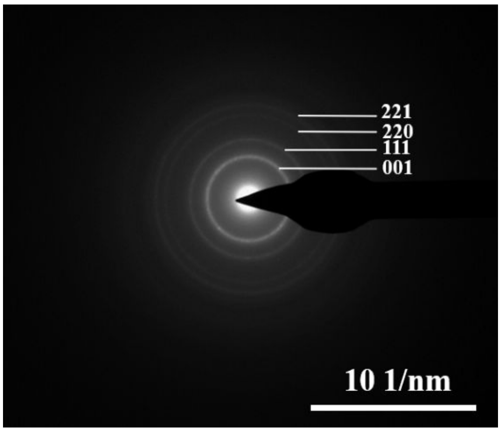

Figure S3. SAED pattern of mesoporous Ag/WO_3 sample, showing the spotty diffraction rings of polycrystalline tungsten oxide wall.

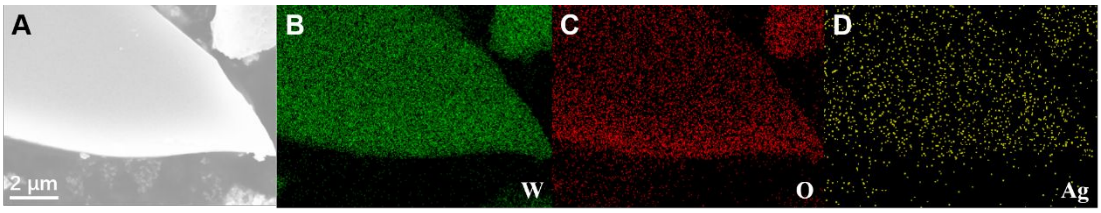

Figure S4. SEM-EDX element mappings images (B, C and D) of W, O and Ag elements of mesoporous Ag/WO_3, revealing the homogeneous distribution of W, O and Ag elements mesoporous Ag/WO_3 materials.

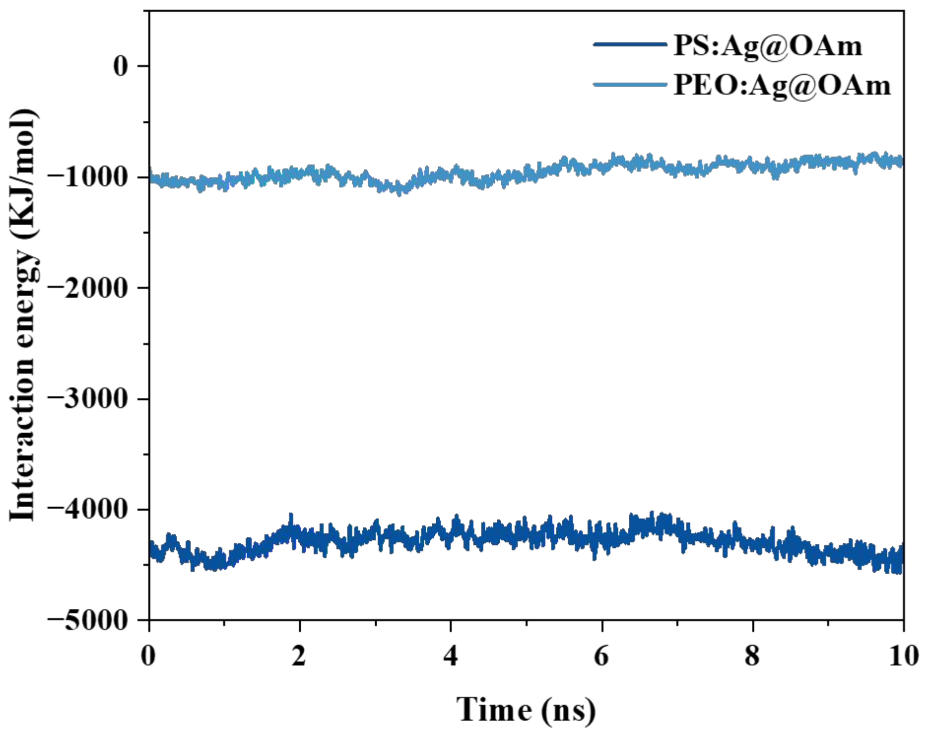

Figure S5. Interaction energies between PEO/PS segments and Ag@OAm. The much higher interaction energy between PS and Ag@OAm than that between PEO and Ag@OAm ensures Ag@OAm well included in PS core of micelle and results in regiospecific co-assembly.

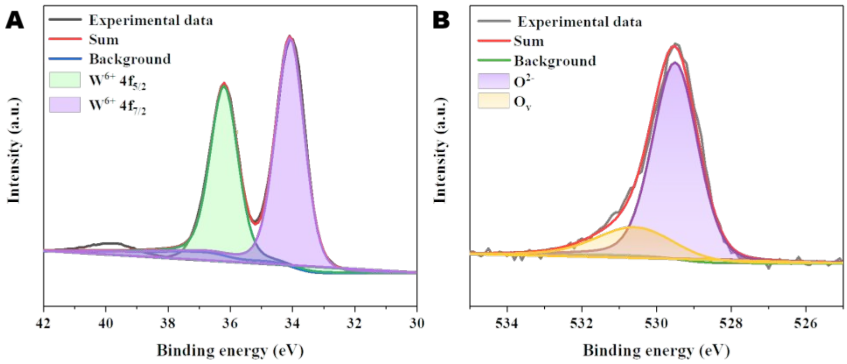

Figure S6. XPS spectra of mesoporous WO_3 in the vicinity of (A) W 4f and (B) O 1s.

The narrow-scan W 4f XPS spectrum of mesoporous WO_3 shows two peaks attributed to W^{6+} $4f_{5/2}$ (36.2 eV) and W^{6+} $4f_{7/2}$ (34.1 eV), respectively, while no peak can be assigned to W^{5+}. The narrow-scan O 1s XPS spectrum of mesoporous WO_3 shows two peaks attributed to oxygen vacancies (531.0 eV) and lattice oxygen O^{2-} (530.4 eV), respectively, and content of oxygen vacancies was calculated to be 21.8%.

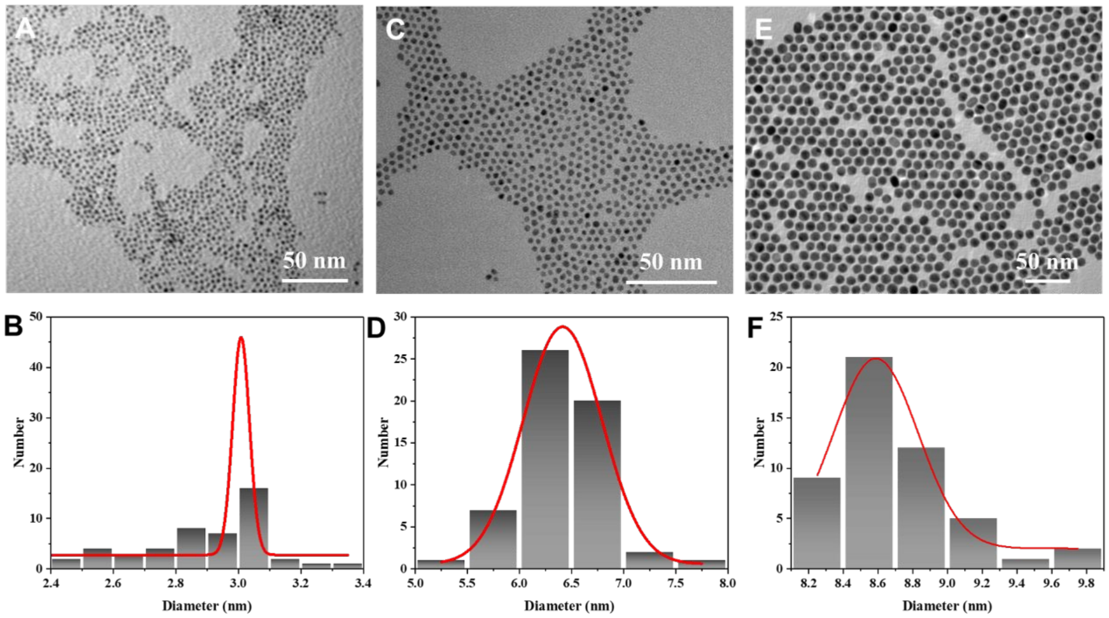

Figure S7. TEM images of (A) Pt NCs, (C) Pd NCs and (E) Au NCs; The particle size distribution profile of (B) Pt NCs, (D) Pd NCs and (F) Au NCs, which indicate that the as-synthesized Pt, Pd and Au NCs possess uniform diameter of 3.0, 6.4 and 8.5 nm, respectively.

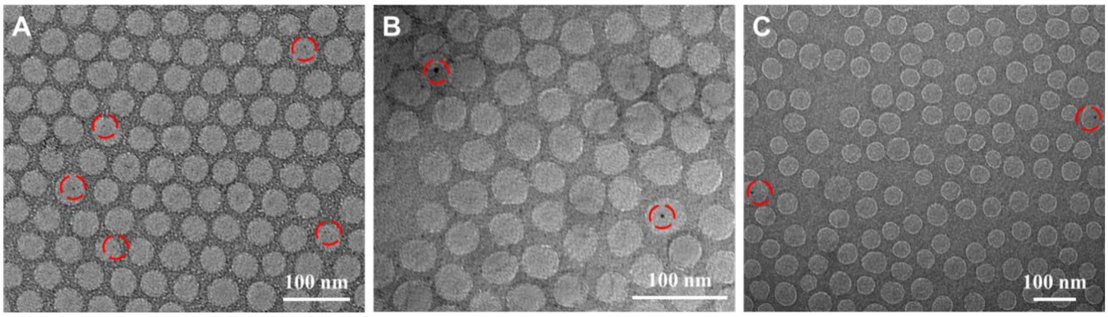

Figure S8. TEM images of TiO_2/PEO-*b*-PS/Pt NCs micelles (A), TiO_2/PEO-*b*-PS/Pd NCs micelles (B) and TiO_2/PEO-*b*-PS/Au NCs micelles (C). The representative nanocrystals encapsulated in PS cores of micelles were highlighted by red circles.

These results reveal that as solvent evaporates, uniform spherical composite micelles can be formed without phase separation via RSCA procedure, and noble metal (Pt, Pd and Au) NCs were encapsulated in the hydrophobic PS core and TiO_2 oligomers were associated with PEO shell of the composite micelles.

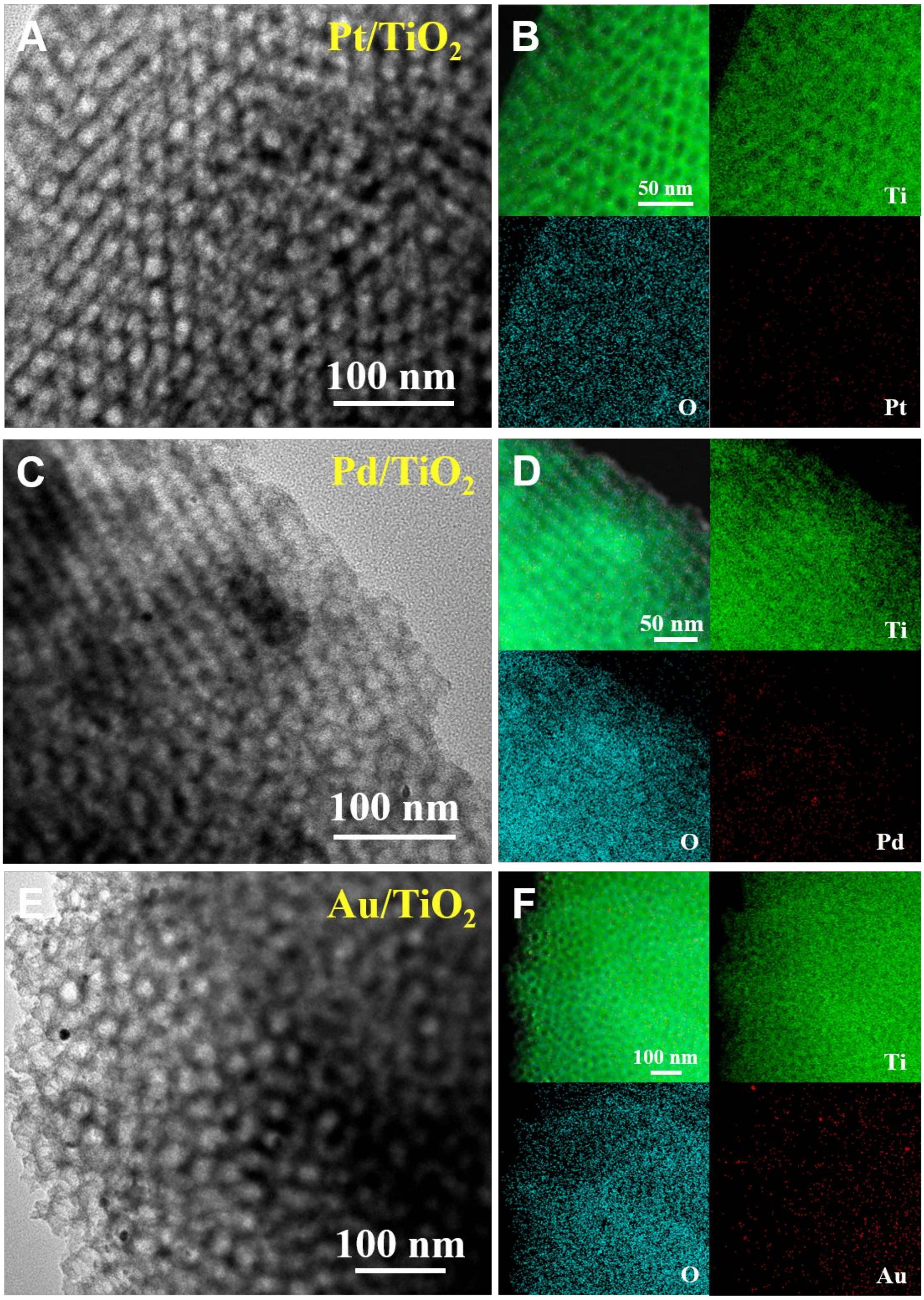

Figure S9. TEM (A, C, E) and the corresponding EDX elemental (Ti, O and noble metal elements) mapping (B, D, F) images of mesoporous Pt/TiO_2, Pd/TiO_2 and Au/TiO_2.

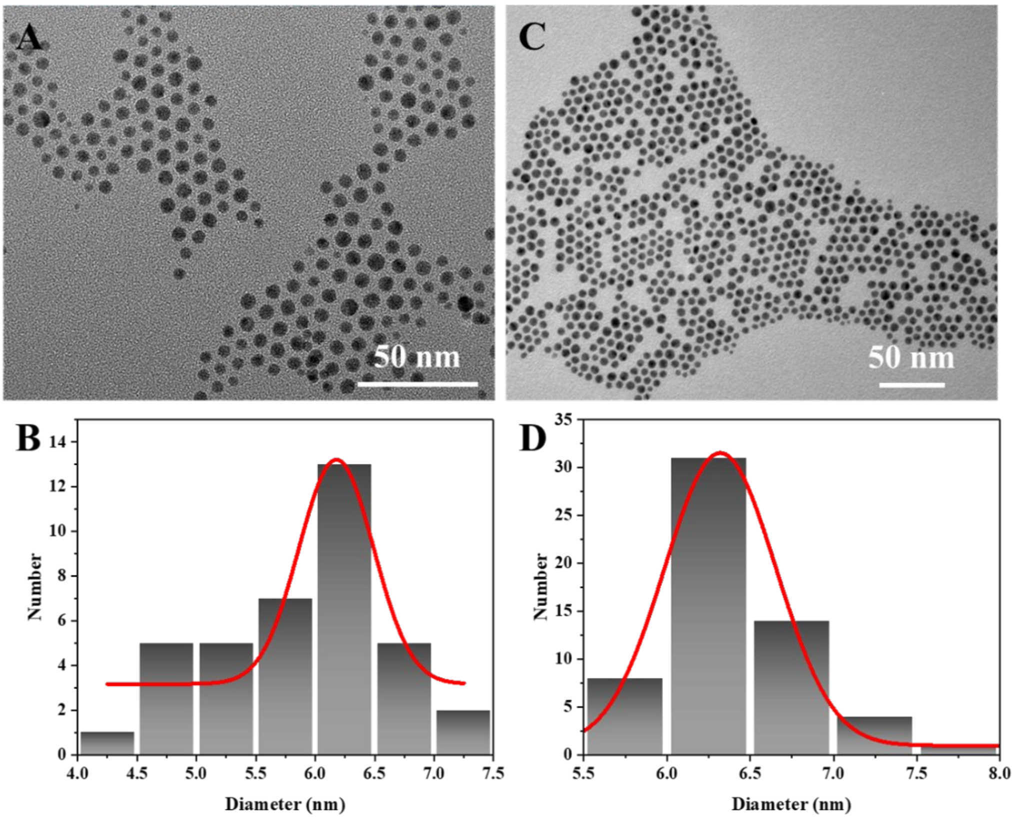

Figure S10. TEM images (A, C) and the particle size distribution profile (B, D) of Au_2Pd_1 and Au_1Pd_1 NCs, showing that the as-synthesized Au_2Pd_1 and Au_1Pd_1 NCs possess uniform diameter of 6.2 and 6.4 nm, respectively.

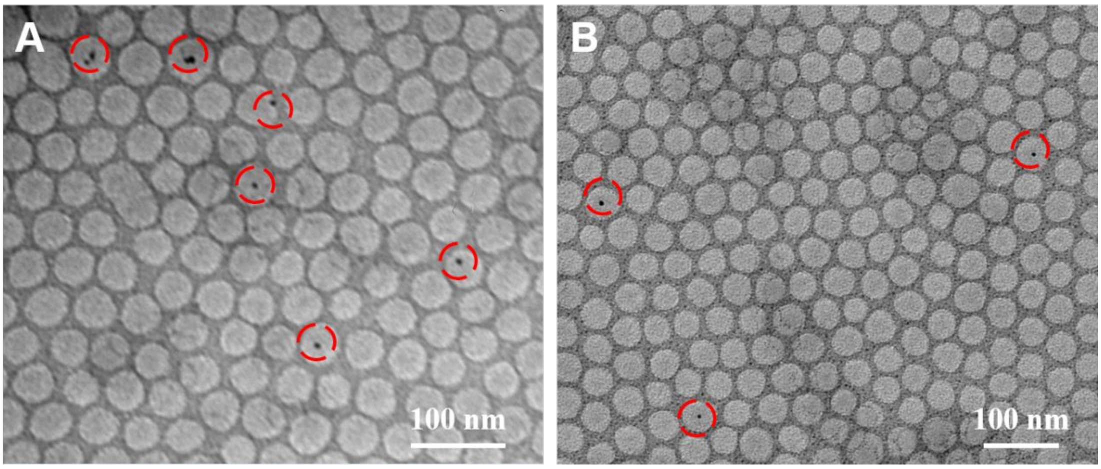

Figure S11. TEM images of TiO_2/PEO-*b*-PS/Au_2Pd_1 NCs micelles (A) and TiO_2/PEO-*b*-PS/Au_1Pd_1 NCs micelles (B). The representative nanocrystals encapsulated in PS cores of micelles were highlighted by red circles.

As solvent evaporates, hydrophilic TiO_2 oligomers, PEO-*b*-PS and hydrophobic alloy NCs can co-assemble simultaneously to form uniform spherical composite micelles without phase separation, and alloy (Au_2Pd_1 and Au_1Pd_1) NCs were well encapsulated in the hydrophobic PS core and TiO_2 oligomers were associated with PEO to form the shell of the composite micelles.

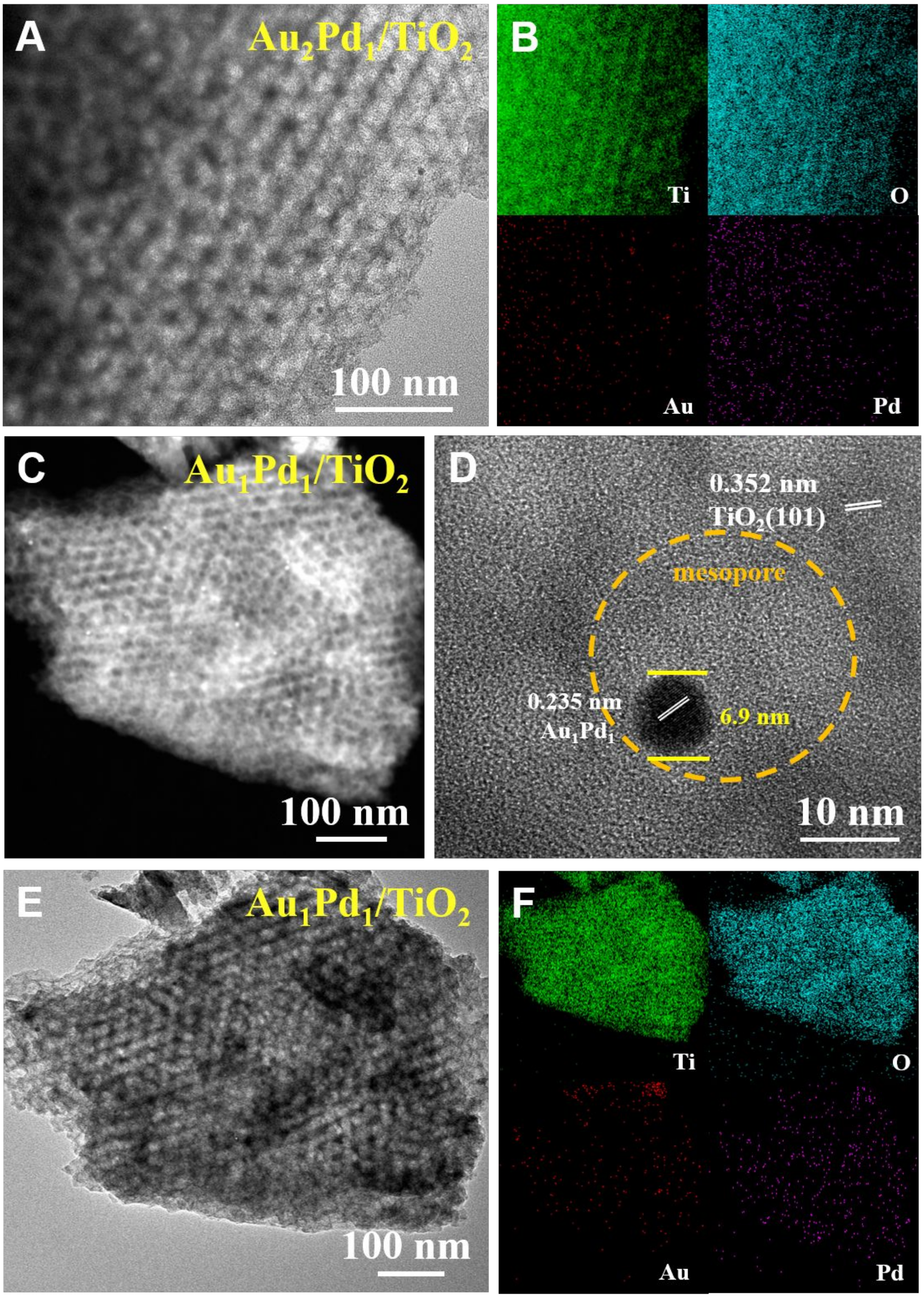

Figure S12. TEM (A, E) and EDX elemental mapping images of Ti, O, Au and Pd elements (B, F) of mesoporous Au_2Pd_1/TiO_2 and Au_1Pd_1/TiO_2 composites, respectively. (C) HADDF-STEM and (D) HRTEM image of mesoporous Au_1Pd_1/TiO_2.

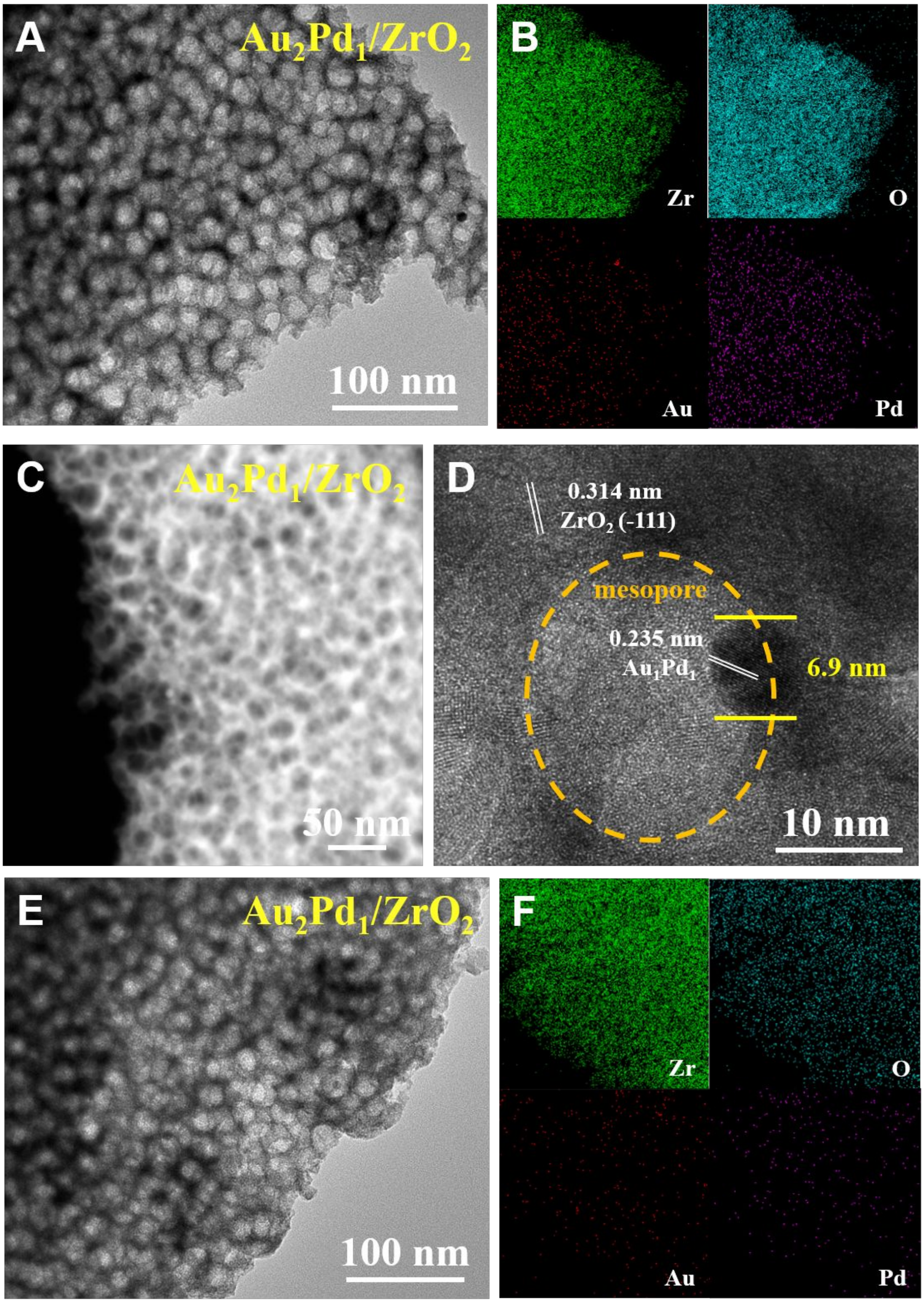

Figure S13. TEM (A, E) and EDX elemental mapping images of Ti, O, Au and Pd elements (B, F) of mesoporous Au_2Pd_1/ZrO_2 and Au_1Pd_1/ZrO_2 composites, respectively. (C) HADDF-STEM and (D) HRTEM image of mesoporous Au_1Pd_1/ZrO_2.

The obtained mesoporous Au_2Pd_1/ZrO_2 and Au_1Pd_1/ZrO_2 via RSCA show that the composites possessed well connected mesopores with alloy NCs anchored on the

inner pore wall without agglomeration (**Figure 4I, S13A, C** and **E**). HRTEM images (**Figure 4J** and **S13D**) show that Au_2Pd_1 NCs of 6.2 nm and Au_1Pd_1 NCs of 6.9 nm were well confined in the uniform mesopores, respectively, and the sizes of these alloy NCs in mesoporous composites is consistent with that of pre-synthesized alloy nanocrystals. The corresponding EDX element mapping images (**Figure S13B** and **F**) indicate the homogeneous distribution of Zr, O, Au and Pd, further demonstrating the successful synthesis of component-customized alloy NCs functionalized mesoporous ZrO_2.

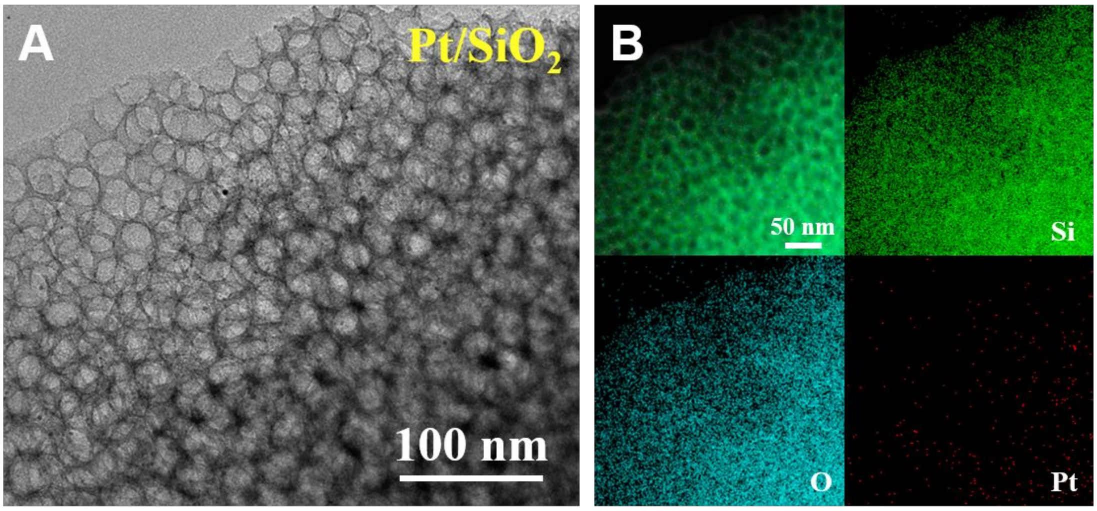

Figure S14. (A) TEM and (B) the corresponding EDX elemental mapping images of Si, O and Pt elements of mesoporous Pt/SiO_2.

The obtained mesoporous Pt/SiO_2 via RSCA exhibit that Pt NCs were homogeneously distributed in mesoprous SiO_2 frameworks (**Figure 4K** and **S14A**). Pt NCs of 3.0 nm were well confined in mesoporous walls of amorphous SiO_2, and the diameter is consistent with that of pre-synthesized NCs (**Figure 4L**), suggesting that this RSCA method can prevent NCs from aggregation validly. The corresponding EDX element mapping images (**Figure S14B**) indicate the homogeneous distribution of Si, O and Pt, further demonstrating the successful synthesis of mesoporous Pt/SiO_2 via RSCA.

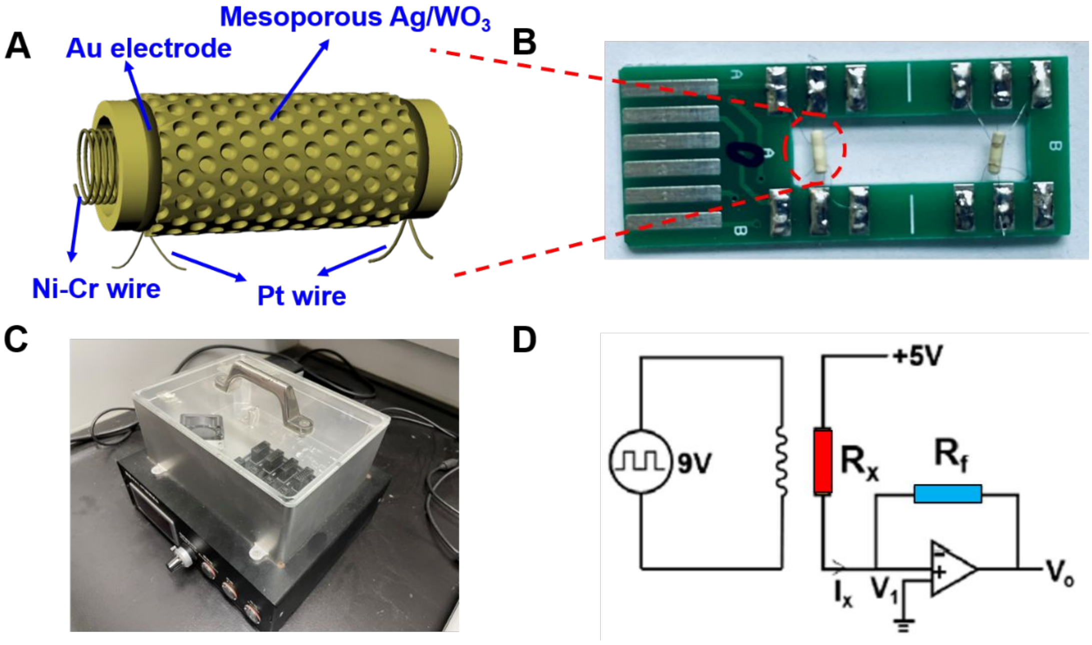

Figure S15. (A) Schematic illustration of the ceramic tube-based sensing device, (B) photo of gas sensors, (C) photo of the gas sensing measurement system and its electric circuit diagram (D).

Ceramic tube-based sensor consists of alumina ceramic tube substrate attached with a pair of Au electrodes, two pairs of Pt wires and a Ni-Cr alloy wire inserted into the tube as a heater (**Figure S15A**). Mesoporous Ag/WO_3 powder was drop-coated on

ceramic tube, and the sensor was welded subsequently on printed circuit board (PCB) for further testing (**Figure S15B**). The electric circuit diagram of gas sensing measurement system is shown in **Figure S15D**, wherein R_x is the resistance of sensing material to be measured, R_f is the reference resistor, I_x and V_o represent the current and output voltage, respectively.

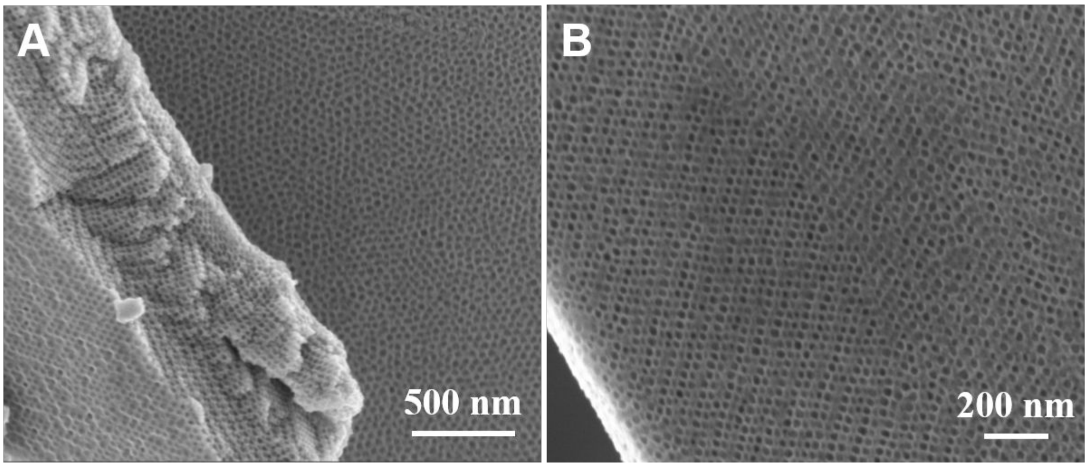

Figure S16. SEM images of mesoporous WO_3, suggesting that the material has highly ordered mesoporous structure.

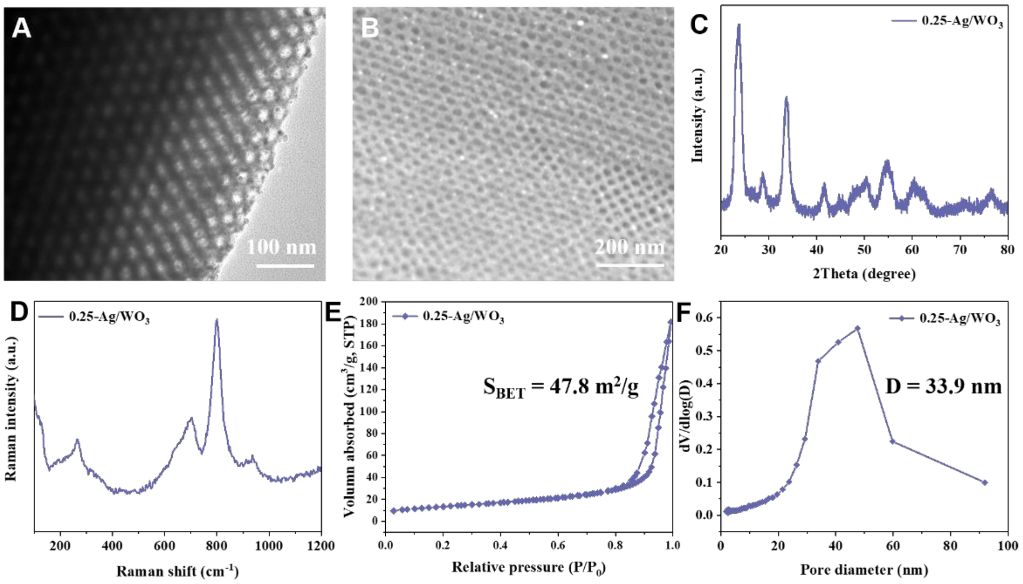

Figure S17. (A) TEM image, (B) SEM image, (C) XRD pattern, (D) Raman spectrum, (E) N_2 adsorption-desorption isotherms and (F) pore sizes distribution of mesoporous 0.25-Ag/WO_3.

TEM and SEM images indicate the ordered mesoporous WO_3 skeleton with highly dispersed 6.5 nm Ag NCs of mesoporous 0.25-Ag/WO_3 (**Figure S17A-B**). The crystalline orthorhombic phase of WO_3 of mesoporous 0.25-Ag/WO_3 can be observed from XRD pattern and Raman spectrum (**Figure S17C-D**), and the absence of

diffraction peaks from metallic Ag confirms the highly dispersed ultrafine Ag NCs, similar to mesoporous 0.5-Ag/WO_3. The mesoporous structure of mesoporous 0.25-Ag/WO_3 was also determined by N_2 adsorption-desorption isotherms analysis, which reveals a high porosity with a specific surface area of 47.8 m^2/g and pore diameter of 33.9 nm (**Figure S17E-F**).

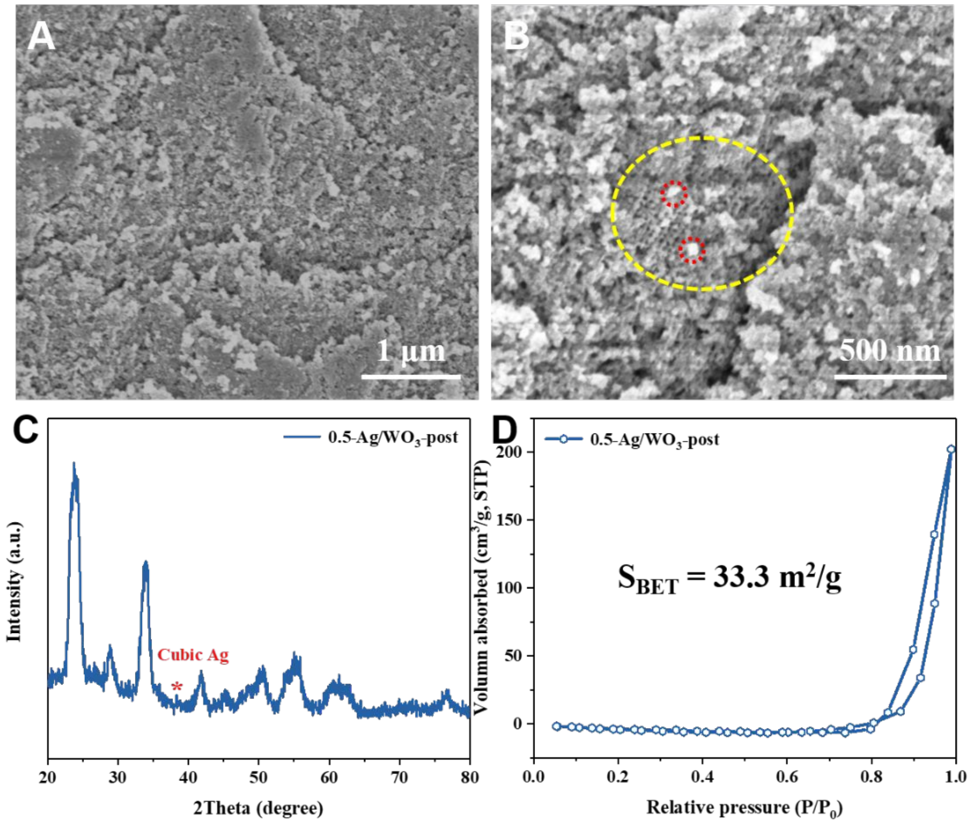

Figure S18. (A-B) SEM images, (C) XRD pattern and (D) N_2 adsorption-desorption

isotherms of 0.5-Ag/WO_3-post. Ag nanoparticles and residual mesoporous structure were highlighted by red and yellow circles, respectively.

SEM images (**Figure S18A-B**) show that 0.5-Ag/WO_3-post have partial blocked and collapsed mesoporous WO_3 frameworks and aggregated Ag particles due to the uncontrollable decomposition of $AgNO_3$ within the porous matrix via the conventional impregnation-reduction method. Notably, diffraction peak at $2\theta = 38.2°$ of cubic Ag (PDF No.04-0783) was detected in 0.5-Ag/WO_3-post materials (**Figure S18C**), implying that 0.5-Ag/WO_3-post synthesized via conventional post-loading method possessed Ag nanoparticles with large diameter. Additionaly, the specific surface area of 0.5-Ag/WO_3-post was calculated to be 33.3 m^2/g (**Figure S18D**), much lower than that of mesoporous WO_3 (51.9 m^2/g) and 0.5-Ag/WO_3 (42.9 m^2/g), which further confirms the collapse of mesoporous WO_3 frameworks resulting from the post-decoration of Ag.

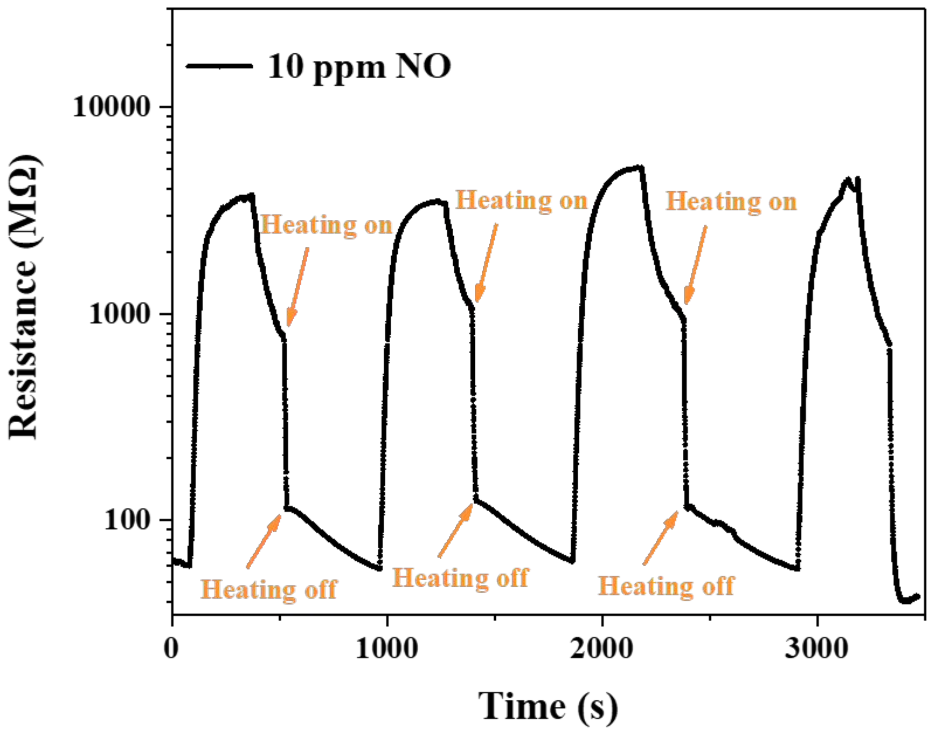

Figure S19. Repeating response and recovery curve of the mesoporous 0.5-Ag/WO_3 sensor based on ceramic tube toward 10 ppm NO at 127 ºC. Mesoporous 0.5-Ag/WO_3 exhibited a retained response of about 78 toward NO of 10 ppm, indicating a reliable NO sensing property.

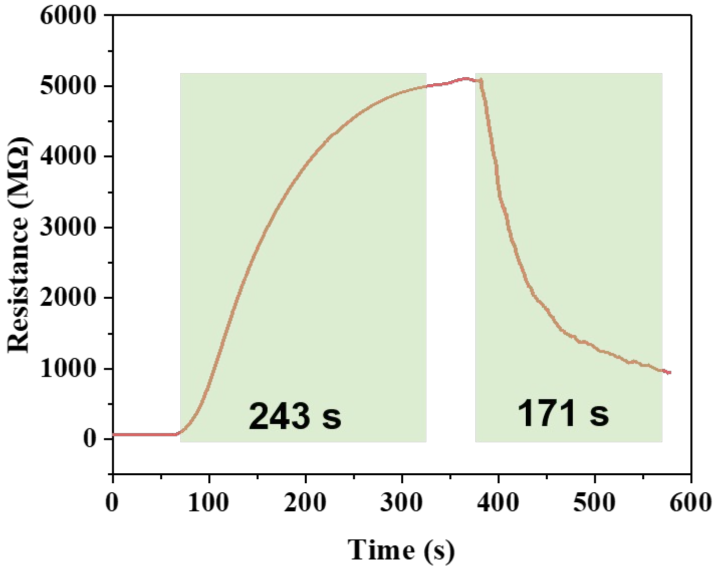

Figure S20. Dynamic response-recovery curve of ordered mesoporous 0.5-Ag/WO_3 sensor based on ceramic tube toward 10 ppm of NO at 127 °C. The response and recovery time of the sensor toward 10 ppm of NO were measured to 243 and 171 s, respectively.

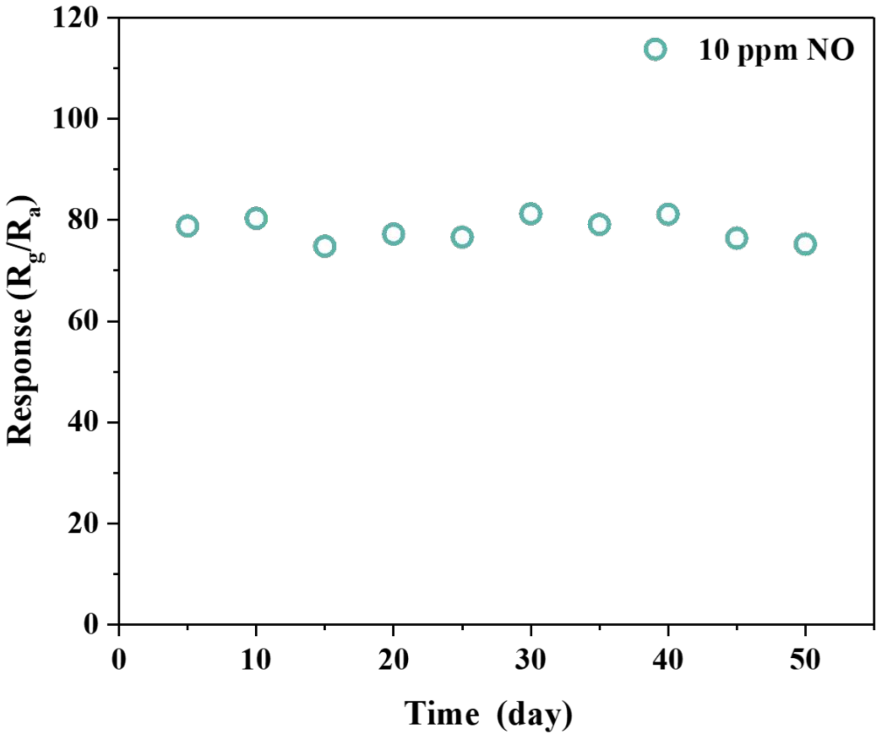

Figure S21. The long-term sensing stability of ordered mesoporous 0.5-Ag/WO_3 sensor fabricated on ceramic tube toward 10 ppm of NO at 127 °C.

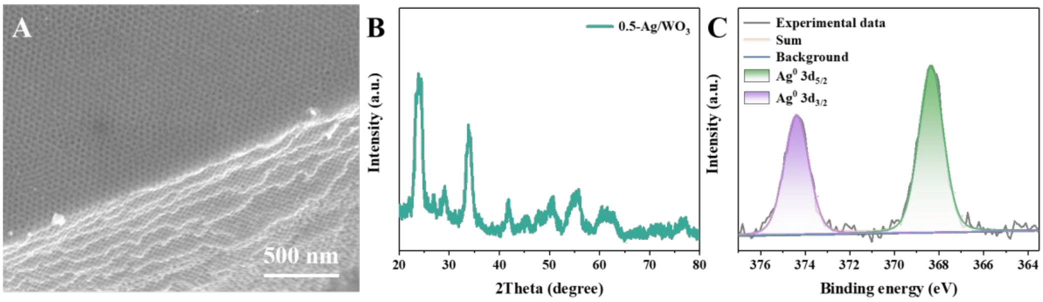

Figure S22. (A) SEM image, (B) XRD pattern and (C) Ag 3d XPS spectra of mesoporous 0.5-Ag/WO_3 material after 2-month sensing stability tests.

SEM image (**Figure S22A**) shows that mesoporous 0.5-Ag/WO_3 materials can retain the ordered mesoporous structure even after 2-month sensing tests. Moreover, no peaks assigned to Ag were detected in XRD pattern 0.5-Ag/WO_3 samples after durability tests (**Figure S22B**), indicating that the well-defined mesoporous structure can effectively prevent Ag NCs from sintering to maintain highly dispersed distribution of Ag and abundant Ag-WO_3 interfaces. Additionally, no peaks belonging to Ag^+ were observed in XPS spectrum (**Figure S22C**). It implies that little Ag was oxidized after the durability tests, and the well-maintained metallic Ag with high catalytic activity for NO oxidization contributed to the stable NO sensitivity.

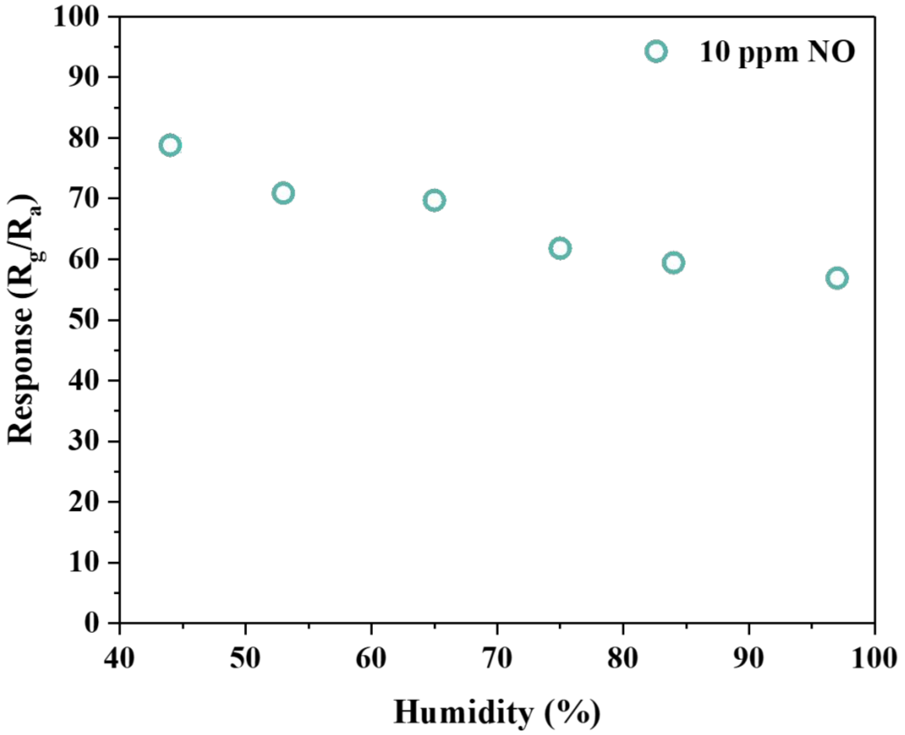

Figure S23. The response of mesoporous 0.5-Ag/WO_3 sensor fabricated on ceramic tube toward 10 ppm of NO at 127 °C under different humidity conditions.

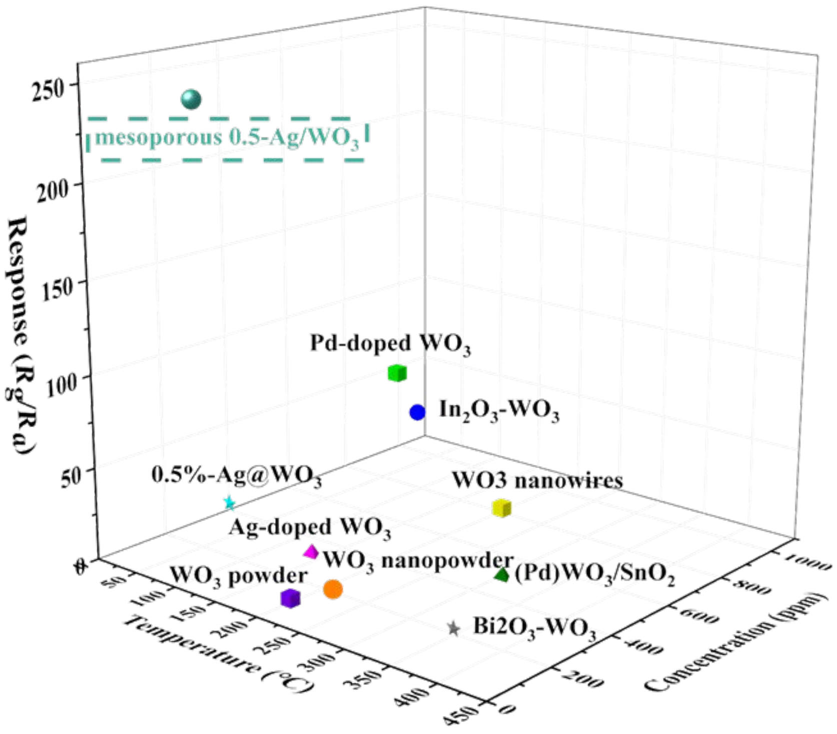

Figure S24. Comparison of the sensing performance of various WO_3-based sensors (detailed information is listed in **Table S3**).

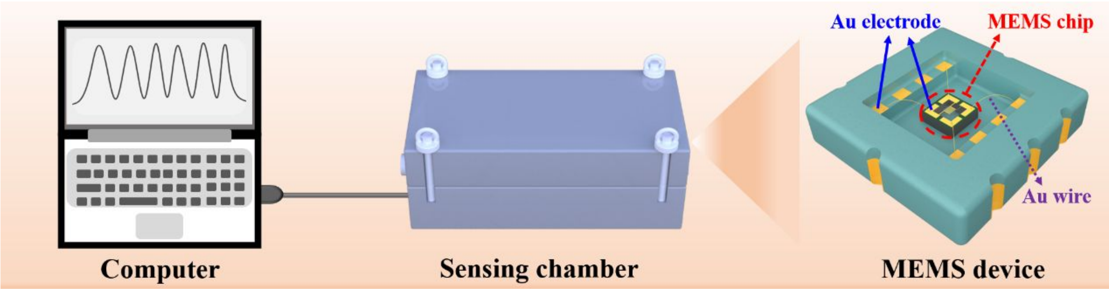

Figure S25. Sketch of the sensing evaluation platform based on MEMS chip.

The MEMS-based sensing evaluation platform mainly consists of a computer for real-time display of dynamical measurement results and a sensing chamber with MEMS chips inside. The MEMS chip (1 × 1 mm^2) is composed of Au interdigitated electrodes to evaluate the resistance of sensing materials and a pair of Au heating electrodes to provide a suitable working temperature controlled by applied voltage.

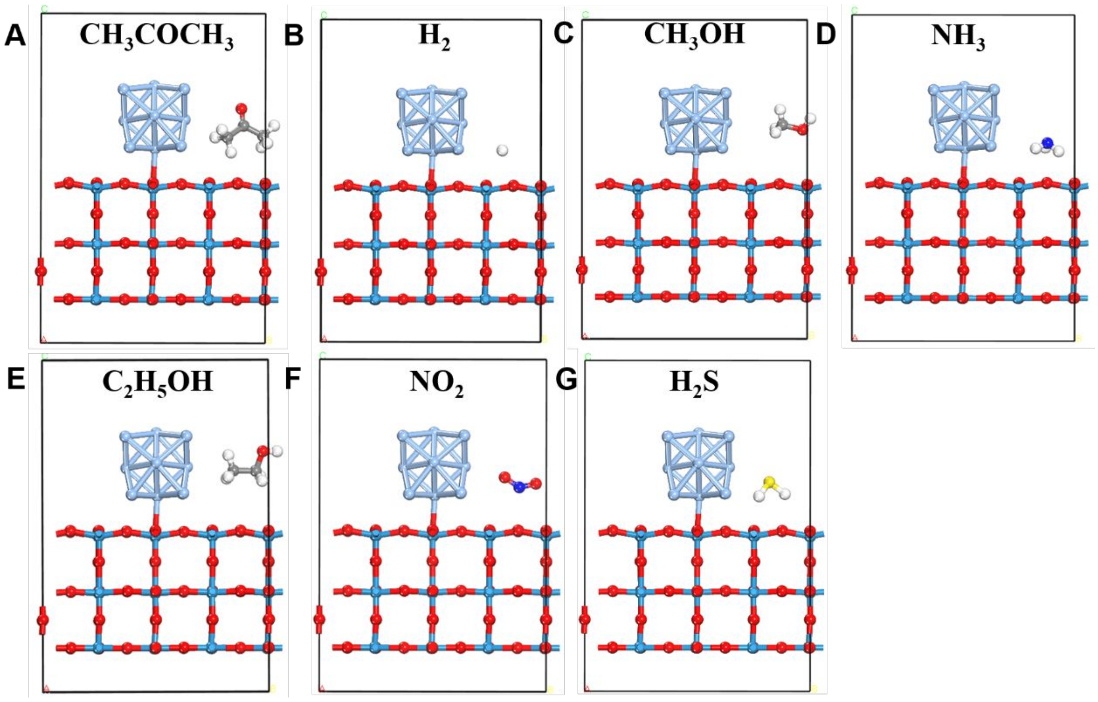

Figure S26. The optimized structure of (A) acetone molecule, (B) hydrogen molecule, (C) methanol molecule, (D) ammonia molecule, (E) ethanol, (F) nitrogen dioxide and hydrogen sulfide molecule absorbed on ordered mesoporous 0.5-Ag/WO_3.

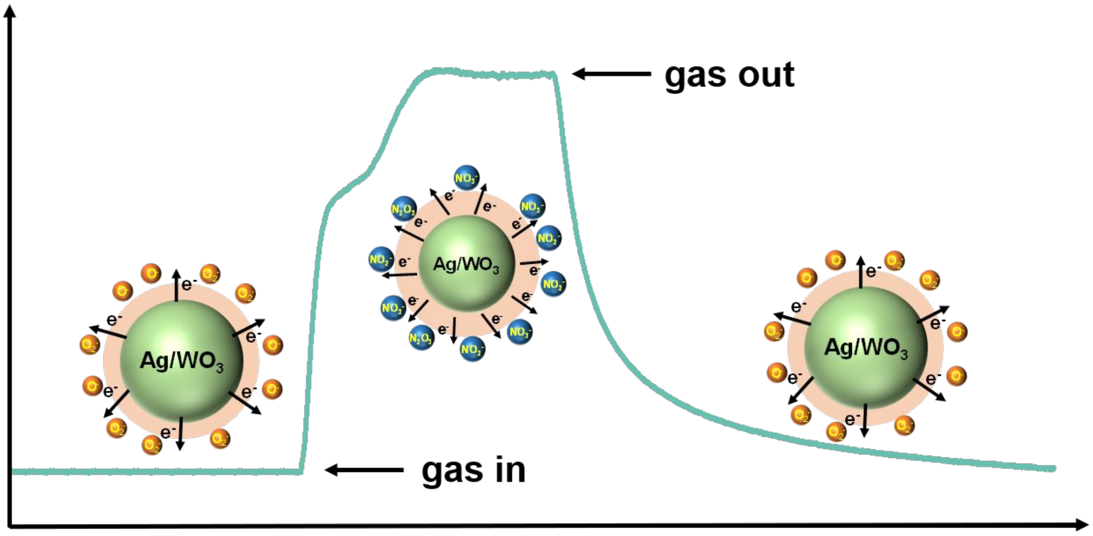

Figure S27. The proposed sensing mechanism of mesoporous Ag/WO_3 to NO.

The NO sensing transients in **Figure S26** were obtained based on dynamic curves collected from the dynamic evaluation system for MEMS sensors. The resultant mild two-step increases of resistance at response period can be ascribed to a re-exposure of sensing material toward the residual NO gas in long pipeline when clean air was purged into the sensing chamber.

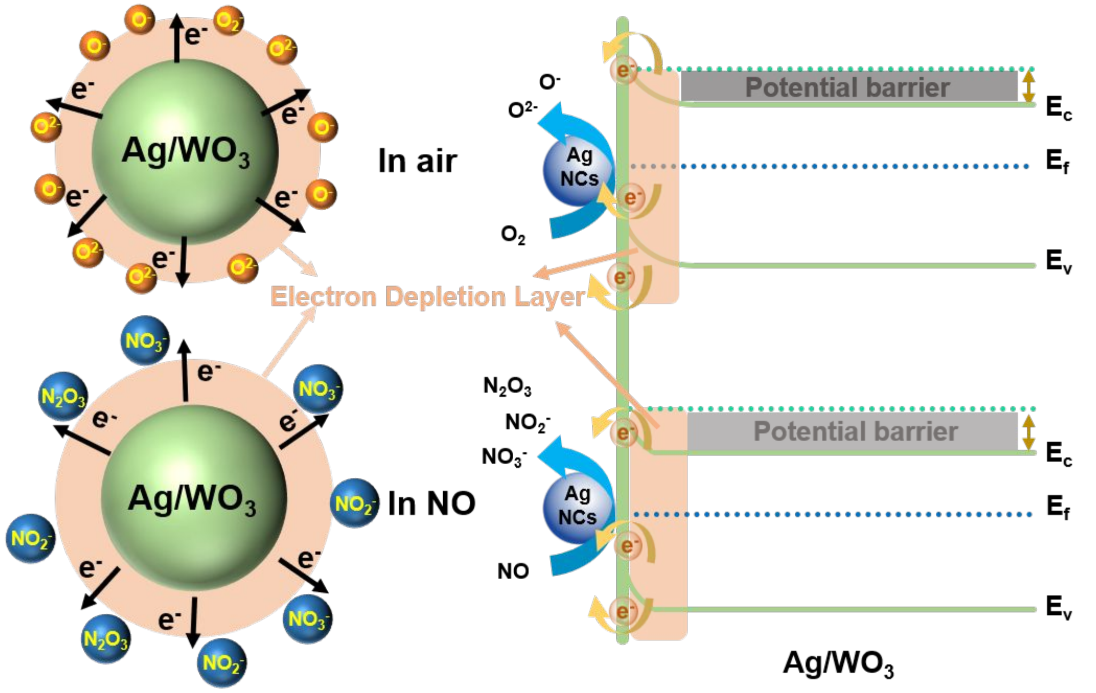

Figure S28. Schematic diagram of energy band structure and electron-transfer process for ordered mesoporous Ag/WO_3 sensitive materials exposed in air and NO-air mixture at high temperature (over 150 °C).

When NO sensing over mesoporous Ag/WO_3 was conducted at temperature over 150 ºC, the reactions between NO gas molecules and active oxygen species of mesoporous Ag/WO_3 during sensing process can be summarized as follows:

$$O_{2\,(gas)} + 4e^- \longrightarrow 2O^{2-}{}_{(ads)} \quad (S1)$$

$$O_{2\,(gas)} + 2e^- \longrightarrow 2O^-{}_{(ads)} \quad (S2)$$

$$NO_{(gas)} + O^-{}_{(ads)} \longrightarrow NO_2^-{}_{(ads)} \quad (S3)$$

$$NO_{(gas)} + O_{2\,(gas)} + e^- \longrightarrow NO_2 + O^-{}_{(ads)} \longrightarrow NO_3^-{}_{(ads)} \quad (S4)$$

$$8NO_2 + 2O^{2-}{}_{(ads)} \longrightarrow 4NO_3^-{}_{(ads)} + 2N_2O_{3\,(ads)} \quad (S5)$$

As active oxygen species exists mainly as O^{2-} and O^- (Equations S1 - S2) at working temperature higher than 150 ºC, mesoporous Ag/WO_3 materials exhibit thick EDL and high resistance in air atmosphere. NO can be oxidized easily to chemisorbed NO_x species with presence of O^{2-} upon materials exposed in NO atmosphere (Equations S3 - S5), while little electron was taken away from sensing material, conducing weak enhancement of EDL thickness and resistance.

Table S1. Textural and gas sensing properties of mesoporous WO_3 and Ag/WO_3 materials.

Sample	BET surface area (m^2/g)	Pore size (nm)	Pore volume (cm^3/g)	W^{5+} content	O_v* content	Response value* (R_g/R_a)
WO_3	51.9	36.2	0.29	0%	21.8%	4.48
0.5-Ag/WO_3	42.9	35.2	0.26	32.7%	24.7%	250.2

O_v: oxygen vacancy, including O^- and O_2^-. The content of W^{5+} and O_v were calculated from corresponding XPS results. Response value: the responses of the sensing devices based ceramic tube substrates were recorded at a working temperature of 127 °C in 25 ppm of NO.

Table S2. Textural properties of hydrophobic noble metal nanocrystals.

Nanocrystals	Size (nm)	Dispersant	Ligand	Concentration (mol/L)
Au	8.5	cyclohexane	oleylamine	0.01
Pt	3.0	cyclohexane	oleylamine	0.002
Pd	6.4	cyclohexane	oleylamine	0.02
Au_2Pd_1	6.2	cyclohexane	oleylamine	0.005
Au_1Pd_1	6.5	cyclohexane	oleylamine	0.005

Table S3. Comparison of NO sensing performance of the sensors based on WO_3 materials with various components and nanostructures.

Materials	Working temperature (°C)	NO concentration (ppm)	Sensitivity (R_g/R_a)	Response/recovery time (s)	Ref.
In_2O_3–WO_3 film	RT	1000	23.9	750/918	19
WO_3 powder	200	100	2.2	--/--	20
WO_3 nanowires	300	500	37	63/88	21
(Pd)WO_3/SnO_2 thick film	400	200	38	20/37.5	22
Pd-doped WO_3 film	200	440	100.3	246/1140	23
0.5%-Ag@WO_3 nanoplates	170	1	55	~20/~50	24
Bi_2O_3-WO_3 thick-film	350	194.2	3.41	138/180	25
Ag-doped WO_3 film	250	40	38.3	--/--	26
WO_3 nanopowder	250	100	15	300/1800	27
Mesoporous 0.5-Ag/WO_3	127	25	250.2	306/178	This work

Table S4. Assignment of *in situ* time resolved DRIFTS absorption bands.

**Wavelength (cm^{-1})**	1216	1270	1430	1586	1615	1658	1866
**NO_x species**	NO_2^-	NO_3^- (monodentate)	NO_3^- (monodentate)	N_2O_3	NO_3^- (bidentate)	NO_3^- (bridged)	N_2O_3

References

[1] Li, Z., Zou, J., Xi, X., Fan, P., Zhang, Y., Peng, Y., Banham, D., Yang, D., Dong, A. Native ligand carbonization renders common platinum nanoparticles highly durable for electrocatalytic oxygen reduction: annealing temperature matters. *Adv. Mater.* **2022**, *34*, 2202743.

[2] Wu, B., Yang, H., Huang, H., Chen, G., Zheng, N. Solvent effect on the synthesis of monodisperse amine-capped Au nanoparticles. *Chin. Chem. Lett.* **2013**, *24*, 457.

[3] Shen, C., Hui, C., Yang, T., Xiao, C., Tian, J., Bao, L. Chen, S., Ding, H., Gao, H. Monodisperse noble-metal nanoparticles and their surface enhanced Raman scattering properties. *Chem. Mater.* **2008**, *20*, 6939.

[4] Zhu, Y., Zhao, Y., Ma, J., Cheng, X., Xie, J., Xu, P., Liu, H., Liu, H., Zhao, H., Wu, M., Elzatahry, A. A., Alghamdi, A., Deng, Y., Zhao, D. Mesoporous tungsten oxides with crystalline framework for highly sensitive and selective detection of foodborne pathogens. *J. Am. Chem. Soc.* **2017**, *139*, 10365.

[5] Anguraj, G., Ashok Kumar, R., Inmozhi, C., Uthrakumar, R., Elshikh, M. S., Almutairi, S. M., Kaviyarasu, K. MnO_2 Doped with Ag Nanoparticles and Their Applications in Antimicrobial and Photocatalytic Reactions. *Catalysts* **2023**, *13*, 397.

[6] Frisch, M. J., Trucks, G. W., Schlegel, H. B., Scuseria, G. E., Robb, M. A.,

Cheeseman, J. R., Scalmani, G., Barone, V., Petersson, G. A. Nakatsuji, H., Wallingford, C. T. *Gaussian 16 revision a. 03.* **2016,** *2*.

[7] Sousa da Silva, A. W., Vranken, W. F. ACPYPE-Antechamber python parser interface. *BMC Res. Notes* **2012,** 5, 1.

[8] Wang, J., Wolf, R. M., Caldwell, J. W., Kollman, P. A., Case, D. A. Development and testing of a general amber force field. *J. Comput. Chem.* **2004**, *25*, 1157.

[9] Abraham, M. J., Murtola, T., Schulz, R., Páll, S., Smith, J. C., Hess, B., Lindahl, E. GROMACS: High performance molecular simulations through multi-level parallelism from laptops to supercomputers. *SoftwareX* **2015**, *1*, 19.

[10] Bussi, G., Donadio, D., Parrinello, M. Canonical sampling through velocity rescaling. *J. Chem. Phys.* **2007**, *126*, 014101.

[11] Parrinello, M., Rahman, A. Polymorphic transitions in single crystals: A new molecular dynamics method. *J. Appl. Phys.* **1981**, *52*, 7182.

[12] Hess, B., Bekker, H., Berendsen, H. J., Fraaije, J. G. LINCS: a linear constraint solver for molecular simulations. *J. Comput. Chem.* **1997**, *18*, 1463.

[13] Darden, T., York, D., Pedersen, L. Particle mesh Ewald: An $N\cdot\log(N)$ method for Ewald sums in large systems. *J. Chem. Phys.* **1993**, *98*, 10089.

[14] Humphrey, W., Dalke, A., Schulten, K. VMD: visual molecular dynamics. *J. Mol. Graph.* **1996**, *14*, 33.

[15] Kresse, G., Furthmuller, J. Efficiency of ab-initio total energy calculations for metals and semiconductors using a plane-wave basis set. *Comput. Mater. Sci.* **1996**, 6, 15-50.

[16] Kresse, G., Hafner, J. Ab initio molecular dynamics for liquid metals. *Phys. Rev. B* **1993**, 47, 558-561.

[17] Perdew, J. P., Burke, K., Ernzerhof, M. Generalized gradient approximation made simple. *Phys. Rev. Lett.* **1996**, 77, 3865-3868.

[18] Perdew, J. P., Burke, K., Ernzerhof, M. Generalized gradient approximation made simple. *Phys. Rev. Lett.* **1997**, 78, 1396.

[19] Chang, B., Wang, C., Lai, H., Wu, R., Chavali, M. Evaluation of Pt/In_2O_3-WO_3 nano powder ultra-trace level NO gas sensor. *J. Taiwan Ins. Chem. E.* **2014**, *45*, 1056.

[20] Akamatsu, T., Itoh, T., Izu, N., Shin, W. NO and NO_2 Sensing Properties of WO_3 and Co_3O_4 Based Gas Sensors. *Sensors* **2013**, *13*, 12467.

[21] Cai, Z., Li, H., Yang, X., Guo, X. NO sensing by single crystalline WO_3 nanowires. *Sensor. Actua. B: Chem.* **2015**, *219*, 346.

[22] Li, H., Cai, Z., Ding, J., Guo, X. Gigantically enhanced NO sensing properties of WO_3/SnO_2 double layer sensors with Pd decoration. *Sensor. Actua. B: Chem.* **2015**, *220*, 398.

[23] Penza, M., Martucci, C., Cassano, G. NO_x gas sensing characteristics of WO_3 thin films activated by noble metals (Pd, Pt, Au) layers. *Sensor. Actua. B: Chem.* **1998**, *50*, 52.

[24] Chen, D., Yin, L., Ge, L., Fan, B. Zhang, R., Sun, J., Shao, G. Low-temperature and highly selective NO-sensing performance of WO_3 nanoplates decorated with silver nanoparticles. *Sensor. Actua. B: Chem.* **2013**, *185*, 445.

[25] Tomchenko, A. A. Structure and gas-sensitive properties of WO_3-Bi_2O_3 mixed thick films *Sensor. Actua. B: Chem.* **2000**, *68*, 48.

[26] Chen, L., Tsang, S. C. Ag doped WO_3-based powder sensor for the detection of NO gas in air. *Sensor. Actua. B: Chem.* **2003**, *89*, 68.

[27] Siciliano, T., Tepore, A., Micocci, G., Serra, A., Manno, D., Filippo, E. WO_3 gas sensors prepared by thermal oxidization of tungsten. *Sensor. Actua. B: Chem.* **2008**, *133*, 321.
